# Dorsal–Caudal and Ventral Hippocampi Target Different Cell Populations in the Medial Frontal Cortex in Rodents

**DOI:** 10.1523/JNEUROSCI.0217-25.2025

**Published:** 2025-04-09

**Authors:** Paola Alemán-Andrade, Menno P. Witter, Ken-Ichiro Tsutsui, Shinya Ohara

**Affiliations:** ^1^Laboratory of Systems Neuroscience, Tohoku University Graduate School of Medicine, Sendai 980-8577, Japan; ^2^Kavli institute for Systems Neuroscience, NTNU Norwegian University of Science and Technology, Trondheim 7491, Norway; ^3^Laboratory of Systems Neuroscience, Tohoku University Graduate School of Life Sciences, Sendai 980-8577, Japan

**Keywords:** dorsal peduncular cortex, hippocampus, longitudinal axis, medial frontal cortex, rodents

## Abstract

Direct projections from the ventral hippocampus (vHPC) to the medial frontal cortex (MFC) play crucial roles in memory and emotional regulation. Using anterograde transsynaptic tracing and ex vivo electrophysiology in male mice, we document a previously unexplored pathway that parallels the established vHPC-MFC connectivity. This pathway connects the dorsal–caudal hippocampus (dcHPC) to specific subregions of the ventral MFC (vMFC), in particular the dorsal peduncular cortex. Notably, this pathway exerts a strong inhibitory influence on vMFC by targeting a substantial proportion of inhibitory neurons. Retrograde transsynaptic tracing in male rats indicated that vMFC subregions project disynaptically back to vHPC. These results, altogether, suggest the existence of a remarkable functional circuit connecting distinct functional areas: the cognition-related dcHPC with the emotion-related vMFC and vHPC. These findings further provide valuable insights in the cognitive and emotional abnormalities associated with the HPC-MFC connectivity in neurological and psychiatric disorders.

## Significance Statement

We reexamined the organization of the circuits connecting the hippocampus (HPC) to the medial frontal cortex (MFC) in rodents. We found that dorsal–caudal HPC (dcHPC) innervates robustly neurons in ventral subregions of MFC, particularly the dorsal peduncular (DP) cortex, including a significant population of inhibitory neurons. Our results show that DP, a subregion critical for emotional and autonomic control, can be modulated by the direct projection from the more cognition-related dcHPC.

## Introduction

Research has shown that interactions between the hippocampus (HPC) and the frontal cortex are essential for learning and memory, and abnormal connectivity between these areas is associated with cognitive and emotional disorders (Godsil et al., 2013; Sigurdsson and Duvarci, 2016). Therefore, understanding the detailed connectivity between HPC and frontal cortex is critical.

Functional neuroimaging studies in humans suggest a topographical organization of HPC–frontal connectivity along the anterior–posterior HPC axis (Robinson et al., 2015; Zeidman and Maguire, 2016; Christiansen et al., 2017). Nonhuman primate studies report unidirectional projections from hippocampal CA1 and subiculum (SUB) to the frontal cortex (Barbas and Blatt, 1995; Insausti and Muñoz, 2001; Aggleton et al., 2015; Wang et al., 2021). Although there is no consensus on which rodent brain region is equivalent to the primate frontal cortex (Uylings et al., 2003; Laubach et al., 2018), the medial frontal cortex (MFC) in rodents, commonly termed the medial prefrontal cortex, receives substantial inputs from HPC (Jay and Witter, 1991; Cenquizca and Swanson, 2007).

Rodent MFC is functionally differentiated along the dorsoventral axis (Heidbreder and Groenewegen, 2003). Dorsal MFC (dMFC) subregions, such as the dorsal anterior cingulate (ACd) and prelimbic (PL) cortices, are involved in attention and working memory (Kesner and Churchwell, 2011), while ventral MFC (vMFC) subregions, including the infralimbic (IL) and dorsal peduncular (DP) cortices, are linked to emotional, autonomic, and sympathetic functions (Gretenkord et al., 2017; Kataoka et al., 2020). Similarly, the rodent HPC has been differentiated in a dorsal HPC supporting spatial cognition and a ventral HPC (vHPC) mediating emotional behaviors (Moser et al., 1993, 1995; Kjelstrup et al., 2002; Strange et al., 2014).

Anatomical studies have shown that the direct HPC-MFC projection originates mainly from vHPC (Jay and Witter, 1991; Cenquizca and Swanson, 2007). Consequently, research has primarily focused on vHPC-MFC connections, which contribute to spatial working memory (Spellman et al., 2015; Abbas et al., 2018), fear memory (Marek et al., 2018; Twining et al., 2020), anxiety (Adhikari et al., 2010; Padilla-Coreano et al., 2016), and social behavior (Phillips et al., 2019; Sun et al., 2020). Electrophysiological experiments further indicate that vHPC targets both excitatory neurons and inhibitory interneurons in MFC (Tierney et al., 2004; Liu et al., 2020; Sánchez-Bellot et al., 2022), in such a manner that vHPC innervation of specific populations of γ-aminobutyric acid (GABA)-ergic neurons in MFC is important for memory (Abbas et al., 2018; Marek et al., 2018), emotional regulation (Padilla-Coreano et al., 2016; Sun et al., 2020), and social behavior (Phillips et al., 2019).

Although the vHPC-MFC circuit is well studied, the caudal part of the dorsal HPC (dcHPC), which includes distal CA1 and proximal SUB, also projects directly to MFC (Jay and Witter, 1991; Cenquizca and Swanson, 2007). Research suggests that dorsal HPC-MFC connectivity is crucial for memory consolidation and learning (Buzsáki, 2015; Maingret et al., 2016; Joo and Frank, 2018). The direct dcHPC-MFC may transmit contextual information processed in dorsal HPC to MFC (Barker et al., 2017) and enhance fear memory reconsolidation (Fukushima et al., 2014; Ye et al., 2017). Despite these potential functions, the structural organization of this pathway remains largely unexplored.

Here, we examined the organization of the dcHPC-MFC circuit using an anterograde transsynaptic tracing method with adeno-associated viral vectors (AAV) and compared it with that of the vHPC-MFC circuit. Our findings showed that dcHPC and vHPC target MFC subregions differently: vHPC mainly targets IL, ventral PL, and medial orbital (MO) cortices, whereas dcHPC predominantly targets the most vMFC subregions, DP and MO cortices. Furthermore, ∼40% of MFC neurons receiving HPC inputs were GABAergic interneurons, with dcHPC innervating a significantly larger proportion of these interneurons than vHPC. Notably, dcHPC preferentially recruited parvalbumin-positive (PV+) interneurons, whereas vHPC targeted PV+ and somatostatin-positive (SOM+) interneurons similarly. Overall, our findings revealed two parallel HPC-MFC circuits, organized along the dorsoventral axis, that differ in their targeting of MFC subregions and engagement of excitatory and inhibitory neurons.

## Materials and Methods

### Animals

All animals were group housed at a 12:12 h reversed day/night cycle and had *ad libitum* access to food and water. The anterograde projection patterns were characterized using adult C57BL/6N mice weighing 20–30 g (*n* = 10 male) and adult SD rats weighing 200–390 g (*n* = 3 male). The retrograde rabies viral tracing experiments were carried out in young adult Wistar rats weighing 200–230 g (*n* = 16 male). For the anterograde transsynaptic experiments, adult C57BL/6N mice (*n* = 31 male) were used. The electrophysiological data were obtained from 8 to 10-week-old male C57BL/6N mice (*n* = 23 male). All animals were purchased from Japan SLC. All experiments were approved by the Center for Laboratory Animal Research, Tohoku University. The experiments were conducted in accordance with the Tohoku University Guidelines for Animal Care and Use.

### Stereotaxic injections for tracer and viral transduction

Animals were anesthetized with isoflurane in an induction chamber and were injected intraperitoneally with ketamine (80 mg/kg) and xylazine (10 mg/kg). Animals were mounted on a stereotaxic frame. Isoflurane was administered via a surgical anesthesia mask throughout the surgery at a stable level between 1 and 2%. Eye ointment was applied to the eyes of the animal to protect the corneas from drying out. The skin overlying the skull was disinfected with iodide, and local anesthesia was injected subcutaneously (lidocaine, 10 mg/kg). The skull was exposed, and a small burr hole was drilled above the injection site. The details of the chemical tracers or viral vectors used in each set of the experiments carried out are summarized in Table 1.

**Table 1.** Overview of viral and nonviral tracer injections across the experiments in this study

Number.	Experiment	Species	Number of animal	Injection site	Viral or nonviral tracer	Survival time (days)	Corresponding figure
1	Conventional anterograde tracing	C57BL/6N mice	10 ♂	HPC	PHA-L and/or BDA	10	Figure 1
2	Electrophysiological experiments	C57BL/6N mice	23 ♂	dcHPC or vHPC	AAV9-CaMKIIa-hChR2(H134R)-EYFP	15	Figure 2
				AMG	AAVrg-hSyn-mCherry		
					or Red Retrobeads IX		
3	Anterograde transsynaptic tracing	C57BL/6N mice	11 ♂	dcHPC or vHPC	AAV1-hSyn-Cre	21	Figure 3
4	Anterograde transsynaptic neuron-tagging	C57BL/6N mice	12 ♂	dcHPC or vHPC	AAV1-hSyn-Cre	21	Figure 4
				MFC	AAV8-hSyn-DIO-mCherry		
5	Anterograde transsynaptic interneuron-tagging	C57BL/6N mice	10 ♂	dcHPC or vHPC	AAV1-hSyn-Cre	15–21	Figure 6
				MFC	AAV9-hDlx-Flex-GFP		
6	Conventional anterograde tracing	Sprague Dawley rats	3 ♂	HPC	PHA-L	10	Figure 7
7	Retrograde tracing with RV for monosynaptic labeling	Wistar rats	5 ♂	dcHPC or vHPC	rHEP5.0-ΔG-mRFP	21	Figure 8*A*,*C*1–3,*E*,*G*
					rHEP5.0-ΔG-AcGFP1		
		Wistar rats	2 ♂	dcHPC or vHPC	rHEP5.0-CVSG-mRFP	2	
					rHEP5.0-CVSG-EGFPx2		
	Retrograde tracing with RV for disynaptic labeling	Wistar rats	9 ♂	dcHPC or vHPC	rHEP5.0-CVSG-mRFP	4–5	Figure 8*B*,*D*,*F*,*H*
					rHEP5.0-CVSG-EGFPx2		

For each experiment, the following details are provided: the species and number of subjects, injection sites, the type of chemical tracer or viral vector used, and survival time. Additionally, the corresponding figure in the Results sections is indicated.

For the conventional anterograde tracing experiments, either 2.5% *Phaseolus vulgaris* leucoagglutinin (PHA-L, Vector Laboratories L-1110) or 3.5% 10 kDa biotinylated dextran amine (BDA, Invitrogen-D1956) was injected by iontophoresis with 12 µA positive current pulses (6 s on, 6 s off) for 10 or 15 min using glass micropipettes, positioned differently along the longitudinal axis of HPC.

For the electrophysiological experiments, AAV9-CaMKIIa-hChR2(H134R)-EYFP (2.2 × 10^12^ GC/ml; 100 nl; Addgene-26969) was injected in either dcHPC or vHPC, and for retrograde labeling, AAVrg-hSyn-mCherry (2 × 10^13^ GC/ml; 2 × 100 nl; Addgene-114472) or, alternatively, Red Retrobeads IX (1:1, 2 × 100 nl; Lumafluor) was injected in the basal amygdala (AMG).

For the anterograde transsynaptic experiments, AAV1-hSyn-Cre (1.9 × 10^13^ to 2.1 × 10^13^ GC/ml; 200 nl; Addgene-10553) was injected in either dcHPC or vHPC, and either AAV8-hSyn-DIO-mCherry (3.6 × 10^12^ GC/ml; 3 × 100 nl; Addgene-50459) or AAV9-hDlx-Flex-GFP (4.4 × 10^12^ GC/ml; 3 × 100 nl; Addgene-83895) was injected along the dorsoventral axis of MFC.

For retrograde transsynaptic tracing, we used recombinant rabies virus (RV) vectors based on HEP-Flury strain (Ohara et al., 2009, 2013). To examine the monosynaptic inputs to the hippocampal subfields, we used a nontranssynaptic G-deleted RV vector [150–300 nl of rHEP5.0-ΔG-mRFP (6.0 × 10^8^ FFU/ml), 150 nl of rHEP5.0-ΔG-AcGFP1 (3.0 × 10^9^ FFU/ml)] or a propagation-competent RV vector [150–300 nl of rHEP5.0-CVSG-mRFP (8.0 × 10^8^ FFU/ml), 150–300 nl of rHEP5.0-CVSG-EGFPx2 (7.0 × 10^8^ FFU/ml)] with 2 d of survival time. Multisynaptic inputs were examined by using RV vectors with longer survival period (4–5 d). Each virus was injected along with 1% of pontamine sky blue in order to mark the injection site.

The stereotaxic coordinates relative to the bregma for mice were (AP, ML, DV) dcHPC, −2.9, +2.1, −1.2 mm; vHPC, −2.9, +3, −3.2 mm; MFC, +2.1, +0.4, (−1, −1.6, −2.3) mm; BLA, −1.1, +3.2, −4.2 mm; BMA, −1.10, +2.8, −4.7 mm, and for rats were dcHPC, −5.1, +4.6, −2.5 mm, and vHPC, −5.5, +6.0, −4.6 mm. For the viral transduction, borosilicate pipettes with 20–30 µm diameter tips were back filled with a volume of 100–200 nl of the AAV vector that was pressure-injected at 50 nl/min using a 1 µl Hamilton microsyringe. The pipette was left in place for additional 10 min, allowing time to diffuse away from the pipette tip before slowly pulling it back from the brain. The wound was sutured and after anesthesia recovery, the animal was returned to its home cage.

### Immunohistochemistry and imaging of neuroanatomical tracing samples

Survival time for injected animals was 10 d after tracer injection and 2–3 weeks after viral injections. Injected animals were deeply anesthetized with isoflurane and perfused intracardially with Ringer's solution (0.85% NaCl, 0.025% KCl, 0.02% NaHCO_3_) followed by the fixative 4% paraformaldehyde (PFA) in 0.1 M phosphate buffer (PB) solution. The brains were then removed from the skulls, postfixed in PFA overnight at 4°C, and then cryoprotected for at least 24 h at 4°C in a 0.125 M PB solution containing 20% glycerol and 2% dimethyl sulfoxide. The brains were cut into 40-µm-thick sections in the coronal plane on a freezing microtome and collected in six equally spaced series for processing.

For immunofluorescence staining, floating sections were rinsed in PB saline (PBS) containing 0.1% Triton X-100 (PBS-Tx), followed by a 1 h incubation in blocking solution containing 5% normal goat serum (NGS) in PBS-Tx at room temperature (RT). Sections were subsequently incubated with primary antibodies diluted in the blocking solution for 20–48 h at 4°C, washed in PBS-Tx (3 × 10 min), and incubated with secondary antibodies diluted in PBS-Tx for 3–5 h at RT. Finally, sections were rinsed in PBS (3 × 10 min), mounted on gelatin-coated slides, air-dried, cleared in xylene, and coverslipped with Entellan New (Merck Chemicals, 107961) mounting medium.

To visualize the PHA-L tracer, we used rabbit anti-PHA-L IgG (1:800, Vector Laboratories AS-2300) as primary antibody and Alexa Fluor 647 goat anti-rabbit IgG (1:400, Jackson ImmunoResearch Laboratories 111-605-144) as secondary antibody. BDA was visualized with Cy3-streptavidin (1:400, Jackson ImmunoResearch Laboratories 016-160-084) added along with the secondary antibodies. The primary antibodies used to visualize protein expression were the following: rabbit anti-Cre (1:3,000, Novagen 69050-3), mouse anti-Cre (1:2,000, Chemicon MAB3120), rat anti-RFP (1:500, ChromoTek AB_2336064), rabbit anti-DsRed (1:500, Takara Bio Z2496N), mouse anti-GAD67 (1:200, Chemicon MAB5406), mouse anti-PV (1:2,000, Sigma-Aldrich P3088), and rat anti-SOM (1:1,000, Millipore Sigma MAB354). The respective secondary antibodies were selected among the following: Alexa Fluor 647 anti-rabbit (1:400, Jackson ImmunoResearch Laboratories 111-605-144), Alexa Fluor 647 anti-mouse (1:400, Jackson ImmunoResearch 115-605-146), Cy3 anti-rat (1:400, Jackson ImmunoResearch 112-165-167), Cy3 anti-rabbit (1:400, Jackson ImmunoResearch Laboratories 111-165-144), and Alexa Fluor 647 anti-rat (1:400, Jackson ImmunoResearch Laboratories 112-605-003). For counterstaining, sections were stained with either guinea pig anti-NeuN (1:1,000, Millipore Sigma ABN90P) or mouse anti-NeuN (1:1,000, Millipore Sigma MAB377) as primary antibodies and either DyLight 405 goat anti-guinea pig IgG (1:400, Jackson ImmunoResearch Laboratories 106-475-003) or Alexa Fluor 488 goat anti-mouse IgG (1:400, Jackson ImmunoResearch Laboratories 115-545-146) as secondary antibodies. Alternatively, NeuroTrace-DeepRed 640/450 (1:200, Invitrogen N21483) was used for counterstaining.

Sections were imaged using an automated scanner (Zeiss Axio Scan Z1) with a 10×/0.45 NA objective. For GAD+ cell quantification, images were scanned across a *Z*-stack range using confocal microscopy (Zeiss LSM-900 with Axio Observer) using a 20×/0.8 NA objective.

### Analysis of neuroanatomical tracing samples

The distribution of labeled axons or postsynaptic neurons in MFC was quantified in coronal sections spaced 240 µm apart. The mouse MFC subregions were identified based on cytoarchitectonic criteria of Van De Werd et al. (2010). The lamination in superficial and deep layers was defined according to Gabbott et al. (1997).

For tracer-labeled fiber quantification, each MFC section was segmented into 100-µm-wide columnar bins delineated along the border between Layers (L)3 and 5. The fluorescence intensity of the labeled fibers within each columnar bin was obtained using ImageJ. The intensity of immunolabeling in all bins was normalized to the bin with maximum intensity in the same sample. To compare the differences in projection patterns distributed across the MFC subregions, we summed up the normalized fluorescence intensities of bins within each subregion. Then, the proportion of labeled fibers in each MFC subregion among the total observed in MFC was calculated for an injection sample, so that values from all subregions sum up to 1 for one individual sample. In figures, individual values are presented along with violin plots. Data in text are given as arithmetic mean ± SEM. The proportions in each subregion were compared using a one-way ANOVA followed by Holm–Šídák multiple-comparison test.

The proportion of Cre-labeled cells per subregion or layer was calculated as the number of Cre-labeled cells in the subregion or layer among the total of Cre-labeled cells in MFC. The proportion of PV+ or SOM+ cells per subregion was calculated as the number of PV+ or SOM+ cells in each subregion among the total of GFP-expressing cells in MFC. Data in figures are presented as individual values in violin plots, including maximum and minimum values and the 25 and 75 percentile range. Data in text are presented as median. The values were compared using Mann–Whitney test corrected for multiple comparisons using the Holm–Šídák method.

The proportion of either GAD+ or GAD− cells per subregion was calculated as the number of GAD+ or GAD− cells in each subregion among the total of mCherry-expressing neurons in MFC. The proportions were compared in each subregion for an injection case using a two-tailed *t* test.

All statistical analyses were carried out using GraphPad Prism 10. Thresholds for significance were set at *p < 0.05, **p < 0.01, ***p < 0.001, and ****p < 0.0001.

### Slice preparation

After 2–3 weeks of viral expression, mice were deeply anesthetized with isoflurane before being killed. After decapitation, brains were rapidly removed and placed in an ice-cold oxygenated cutting solution containing the following (in mM): 110 choline-Cl, 2.5 KCl, 7 MgCl_2_, 0.5 CaCl_2_, 25 glucose, 1.25 NaH_2_PO_4_, 25 NaHCO_3_, 11.5 Na-ascorbate, 3 Na-pyruvate, and 100 D-mannitol. Coronal slices of MFC (300 µm thick) were cut on a vibratome (DSK Linearslicer PRO 7) keeping the brain immersed in ice-cold cutting solution. Then, slices were transferred to artificial cerebrospinal fluid (ACSF) containing the following (in mM): 126 NaCl, 3 KCl, 1.2 NaH_2_PO_4_, 10 glucose, 26 NaHCO_3_, 3 MgCl_2_, and 0.5 CaCl_2_, bubbled with O_2_. Slices were kept for 30 min at 35°C, before being allowed to recover for at least 30 min at RT. All chemicals were obtained from Sigma-Aldrich or Wako Pure Chemical Industries.

### Electrophysiology

Recordings were conducted at 28–30°C in oxygenated ACSF containing the following (in mM): 126 NaCl, 3 KCl, 1.2 NaH_2_PO_4_, 10 glucose, 26 NaHCO_3_, 1.5 MgCl_2_, and 1.6 CaCl_2_ (∼295 mOsm). For each individual slice, pyramidal neurons located in MFC subregions were identified by infrared-differential interference contrast microscopy. AMG-projecting neurons were selected for recording by the retrobeads label or the mCherry expression, result from retrograde spread, observed under fluorescent illumination. Whole-cell voltage–clamp recordings were performed using borosilicate glass pipettes (2–4 MΩ) filled with a Cs-based intracellular solution containing the following (in mM): 120 Cs-gluconate (Hellobio, HB4822), 10 HEPES, 10 Na-phosphocreatine, 4 Mg-ATP, 0.4 Na-GTP, 0.5 EGTA, 2 QX314, and 10 TEA-chloride, pH ∼7.3 (CsOH; ∼290 mOsm). Biocytin (2.5%) was included in the internal solution, which was allowed to diffuse throughout the cell for posterior confirmation of location by immunolabeling. Electrophysiological recordings were made with a double integrated patch amplifier and SutterPatch software (Sutter Instrument), filtered at 2 kHz and sampled at 10 kHz. The initial series resistance was <20 ΩM, and recordings ended if series resistance rose >25 ΩM. Excitatory and inhibitory postsynaptic currents (EPSC and IPSC) were recorded at −70 and +10 mV, respectively. In some experiments, to isolate the monosynaptic inputs, TTX (1 µM), 4-AP (100 µM), and elevated Ca^2+^ (4 mM) were included in the recording solution, a mix that blocks the action potentials but restores presynaptic release (Little and Carter, 2012; Liu and Carter, 2018). In other experiments, the excitatory responses were isolated by including gabazine (10 µM) in the recording solution to block the GABA_A_ receptors. All chemicals were obtained from Sigma-Aldrich or Wako Pure Chemical Industries.

### Optogenetic stimulation

Channelrhodopsin-2 (ChR2) was expressed in presynaptic neurons, and their terminal axons in MFC were activated with a brief light pulse from a blue LED (473 nm, Zeiss Colibri 7). For wide-field illumination, light was transmitted via a 40×/0.8 NA objective. For each experiment, LED pulse power and duration were adjusted to a typical value of 15 mW and 2 ms, respectively.

### Immunohistochemistry and imaging of recorded slices

Slices containing biocytin-filled cells were fixed in 4% PFA overnight at 4°C. For staining, slices were washed with PBS-Tx (0.3%; 5 × 15 min), then incubated in 10% NGS blocking solution in PBS-Tx for 3 h at RT, and then incubated in the same blocking solution containing a diluted mixture of primary antibodies at 4°C for 4 d. Subsequently, slices were washed with PBS-Tx (0.3%; 5 × 15 min) and incubated in a dilution of secondary antibodies diluted in PBS-Tx overnight at RT. Slices were then washed in PBS-Tx (3 × 15 min) and stored in PBS at 4°C. Slices were dehydrated by subsequent 10 min exposure to ethanol/distilled water mixtures of 30, 50, 70, and 90%, then two times of 100% ethanol, and a (1:1) mixture of ethanol/methyl salicylate. Slices were then cleared with methyl salicylate and embedded on metal slides with cover glass. Dehydration and clarification were not applied when using RetroBeads as a tracer. *Z*-stack images were collected with confocal microscope (Zeiss LSM-900 with Axio Observer) using a 20×/0.8 NA objective.

GFP expression was enhanced with the primary antibody chicken anti-GFP (1:500, Abcam AB13970) and secondary antibody Alexa Fluor 488 anti-chicken (1:500, Invitrogen A-11039). mCherry expression was enhanced using primary antibody rabbit anti-DsRed (1:300, Takara Bio Z2496N) and secondary antibody Cy3 anti-rabbit (1:200, Jackson ImmunoResearch Laboratories 111-165-144). Biocytin was visualized with Alexa Fluor 647 streptavidin (1:200, Jackson ImmunoResearch Laboratories 016-600-084) added to secondary antibody mix. For counterstaining, the primary antibody used was guinea pig anti-NeuN (1:500, Millipore Sigma ABN90) and secondary antibody DyLight 405 anti-guinea pig (1:200, Jackson ImmunoResearch Laboratories).

### Analysis of electrophysiological data

EPSC and IPSC events were analyzed off-line using the Synaptic Event Analysis module from the SutterPatch application (Igor Pro software). Quantitative data from multiple slices are given as median and 25th and 75th percentile. Data in figures are presented as individual values indicating median in violin plots showing minimum and maximum values, with 25–75 percentile range. Data in text are only given as medians. Statistical analysis was performed using the GraphPad Prism 10 software. Mann–Whitney test was used and corrected for multiple comparisons using the Holm–Šídák method. Thresholds for significance were set at *p < 0.05, **p < 0.01, ***p < 0.001, and ****p < 0.0001.

## Results

### dcHPC and vHPC project differently to MFC subregions in mice

**Figure 1.**
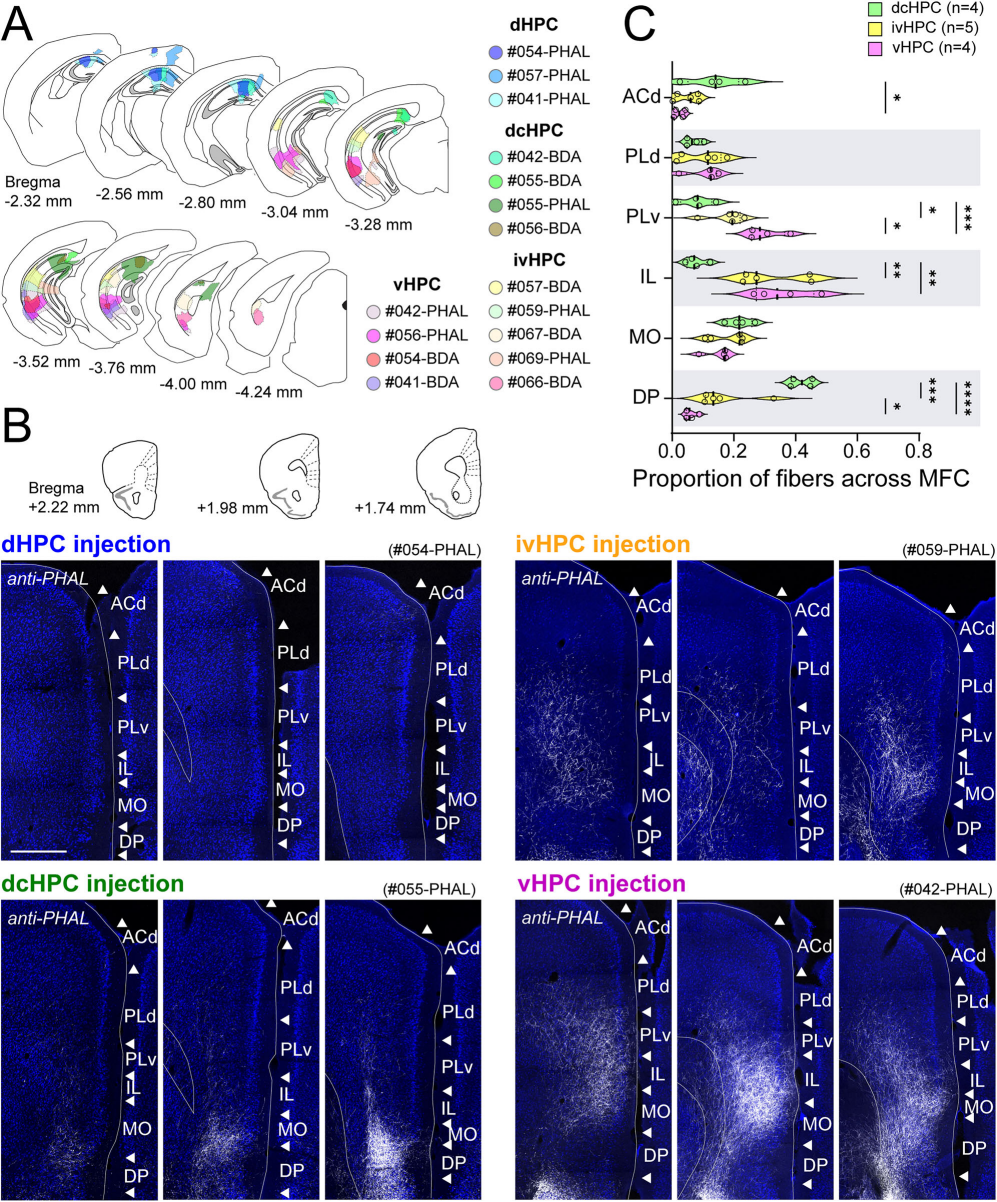
Projections originating along the dorsoventral axis of HPC, visualized by anterograde tracer injections, are differently distributed across MFC subregions in mice. ***A***, Summary of anterograde tracer (BDA or PHA-L) injection sites along the dorsoventral axis of the mouse HPC (10 males) displayed in coronal sections. The HPC injection level was differentiated into dHPC, dcHPC, ivHPC, and vHPC. ***B***, Representative samples of anterogradely labeled fibers in MFC (at three coronal levels as shown in the top inset) for injections in dHPC (case #054-PHAL), dcHPC (case #055-PHAL), ivHPC (case #059-PHAL), or vHPC (case #042-PHAL). Scale bar, 500 µm. ***C***, Quantification of the proportion of labeled fibers across MFC subregions for injections in dcHPC (green, *n* = 4), ivHPC (yellow, *n* = 5), and vHPC (pink, *n* = 4). Each circle represents the proportion of labeled fibers in the MFC subregion among the total observed in MFC for one injection. Values from all subregions sum up to 1 for one individual sample. Data are presented as violin plots. One-way ANOVA, followed by Holm–Šídák's multiple-comparison test: ****p < 0.0001; ***p < 0.001; **p < 0.01; *p < 0.05.

To reexamine the projections from HPC to MFC, we injected anterograde tracers PHA-L or BDA along the longitudinal axis of HPC in mice (10 males; Fig. 1). According to their dorsoventral position in HPC, the injection sites were clustered in four groups: the rostral part of the dorsal portion of HPC (dHPC, *n* = 3), the dcHPC (*n* = 4), the intermediate to ventral levels of HPC (ivHPC, *n* = 5), and the vHPC (*n* = 4; Fig. 1*A*). Though in general, projections from HPC innervated caudal portions of MFC more densely, the four groups presented characteristic projection patterns to different subregions of MFC. Injections in dHPC, predominantly located in the proximal CA1 of anterior sections of HPC, did not result in any clear, distinguishable label in MFC (Fig. 1*B*, top-left). This is in line with previous reports (Jay and Witter, 1991; Cenquizca and Swanson, 2007). Injections in dcHPC, involving the distal portion of CA1 and the proximal portion of SUB, resulted in dense labeling in all layers of DP and MO, spreading lightly to deep layers of IL, PL, and ACd (Fig. 1*B*, bottom-left). This particular topographical organization of the projections from dcHPC was not clearly described in previous studies (Jay and Witter, 1991; Cenquizca and Swanson, 2007). Injections in ivHPC, located more ventrally, mainly involving intermediate parts of CA1, resulted in labeled fibers distributed densely throughout all layers of MO and IL, moderately to the ventral part of PL (PLv) and DP, spreading to deep layers of the dorsal part of PL (PLd; Fig. 1*B*, top-right). Finally, injections in vHPC involved more ventral and posterior sections of field CA1 and in some cases included ventral SUB. The projection pattern observed following these vHPC injections was consistent with previous studies, with fibers spreading densely into all layers of MO, IL, and PLv, and moderately into deep layers of DP and PLd (Fig. 1*B*, bottom-right, Jay and Witter, 1991; Cenquizca and Swanson, 2007).

Since the distribution patterns did not differ between the samples with pure CA1 injection and that with CA1 + SUB injection, these samples were grouped together in the following analysis. To verify the differences in the organization of these parallel HPC-MFC pathways, we calculated the proportion of labeled fibers detected in each MFC subregion among the total observed in MFC (Fig. 1*C*) and analyzed whether the projection patterns from distinct levels of HPC differed significantly in the targeted MFC subregions. In view of the sparse labeling observed in MFC from dHPC injections (Fig. 1*B*, top-left), this group was excluded from further analysis. The results showed that PLd and MO subregions were targeted similarly by all projecting HPC levels (for PLd, vHPC vs ivHPC, *p* = 0.79; vHPC vs dcHPC, *p* = 0.77; ivHPC vs dcHPC, *p* = 0.77; for MO, vHPC vs ivHPC, *p* = 0.48; vHPC vs dcHPC, *p* = 0.22; ivHPC vs dcHPC, *p* = 0.48; one-way ANOVA followed by Holm–Šídák's multiple-comparison test). Both IL and PLv received stronger innervations from ivHPC and vHPC (for IL, vHPC vs ivHPC, *p* = 0.63; vHPC vs dcHPC, *p* = 0.004; ivHPC vs dcHPC, *p* = 0.004; for PLv, vHPC vs ivHPC, *p* = 0.02; vHPC vs dcHPC, *p* = 0.0008; ivHPC vs dcHPC, *p* = 0.02). In contrast, ACd and DP received denser innervation from dcHPC (for ACd, vHPC vs ivHPC, *p* = 0.49; vHPC vs dcHPC, *p* = 0.046; ivHPC vs dcHPC, *p* = 0.08; for DP, vHPC vs ivHPC, *p* = 0.03; vHPC vs dcHPC, *p* < 0.0001; ivHPC vs dcHPC, *p* = 0.0003). Together, these results revealed that although dorsal-to-ventral HPC levels innervate all MFC subregions, projections from HPC to MFC are organized along the dorsoventral axis. Injections in ivHPC presented a projection pattern quite alike vHPC injections with the IL cortex as their main target. Conversely, dcHPC injections presented a significant difference in main targeted MFC subregions, heavily innervating the more ventrally located DP cortex and less strongly ACd. Thus, in the subsequent sections, we will focus on comparing the parallel pathways from dcHPC and vHPC to MFC.

### dcHPC and vHPC elicit excitatory and inhibitory responses in projection neurons in the mouse MFC

**Figure 2.**
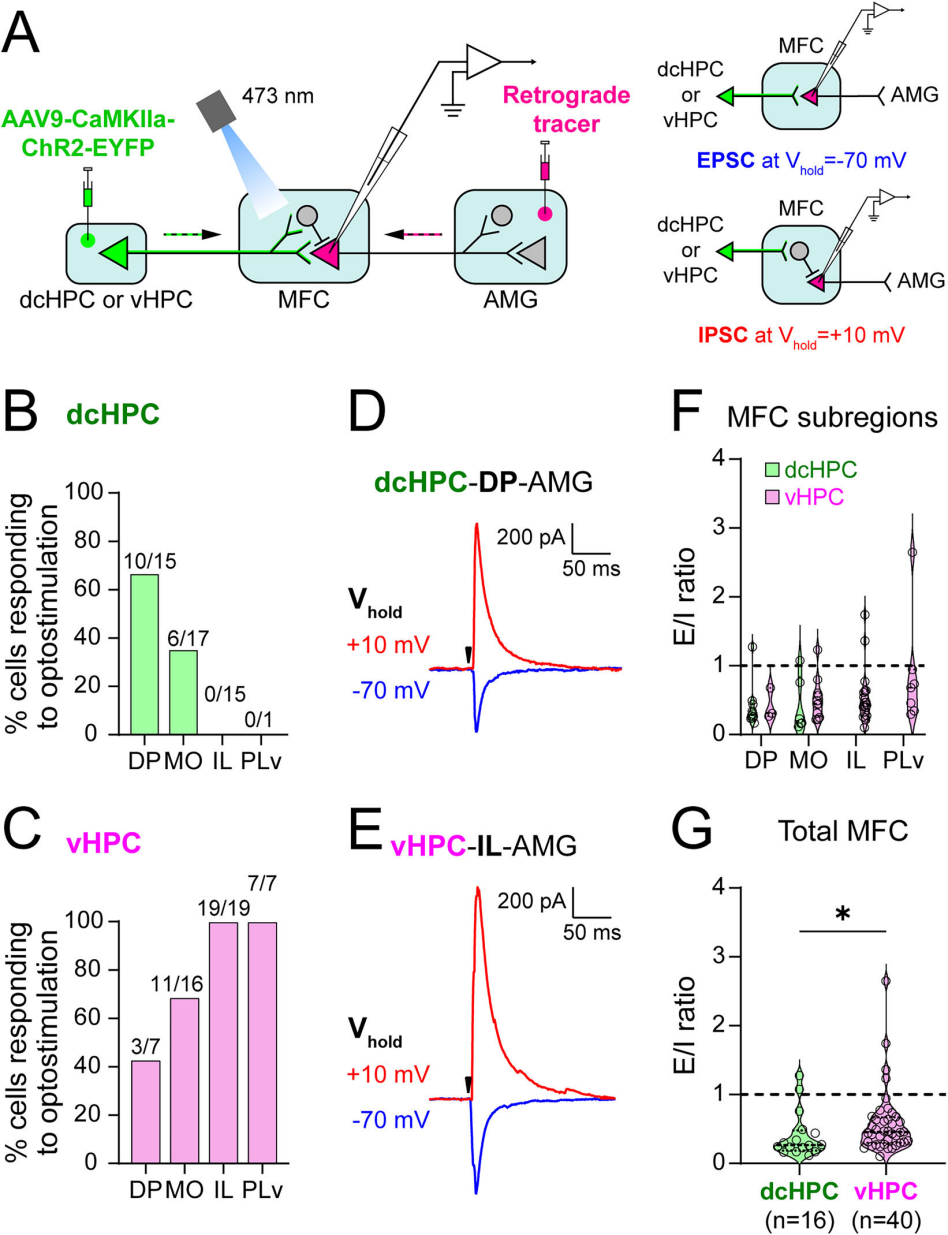
dcHPC and vHPC connect neurons in different subregions in the mouse MFC. ***A***, Left, The general scheme of the electrophysiological experiment: AAV9-CaMKIIa-ChR2-EYFP was injected in either dcHPC or vHPC to express ChR2 in projection neurons. In parallel, the retrograde AAVrg-hSyn-mCherry, or in some cases Red Retrobeads, was injected in AMG to label AMG-projecting neurons in MFC. Whole-cell voltage–clamp recordings were carried out in acute MFC coronal slices from AMG-projecting neurons (rose-colored) while optically stimulating the dcHPC or vHPC axon terminals in MFC. Recorded neurons were labeled with biocytin for posterior localization. Right, Strategy used to record MFC excitatory input at holding potential of −70 mV (EPSC, top) or inhibitory input (IPSC, bottom) mediated via local interneurons at holding potential of +10 mV. In the schemes, principal neurons and interneurons are represented by triangles and circles, respectively. ***B***,***C***, Proportion of responding cells to optical stimulation of dcHPC (***B***) or vHPC (***C***) axon terminals across the MFC subregions. ***D***, Responses from an AMG-projecting cell in DP to light-evoked dcHPC inputs at holding potential of −70 mV (EPSC, blue) and +10 mV (IPSC, red). The black arrow indicates light pulse. ***E***, Responses from an AMG-projecting cell in IL to light-evoked vHPC inputs at holding potential of −70 mV (EPSC, blue) and +10 mV (IPSC, red). The black arrow indicates light pulse. ***F***, Summary of the ratio of light-evoked responses recorded at −70 and +10 mV presented as an E/I ratio. Higher values mean the input is biased to excitation whereas lower values toward inhibition. Cells receiving inputs from dcHPC and vHPC are presented in green and pink, respectively. Data are shown as violin plots, where each circle represents one cell. No significant differences were found across the MFC subregions (*p* = 0.1, Kruskal–Wallis test). ***G***, Summary of the light-evoked E/I ratios pooled from all recorded cells in MFC for dcHPC (green, *n* = 16 cells) or vHPC inputs (pink, *n* = 40 cells). Data are presented as violin plots, where each circle corresponds to one cell. Two-tailed Mann–Whitney test: *p < 0.05.

Projections from the different MFC subregions to AMG are thought to be important for the modulation of anxiety- and fear-related behaviors (Sierra-Mercado et al., 2011; Marek et al., 2013; Adhikari et al., 2015; Borkar et al., 2024). Inputs from HPC seem to be able to modulate the activity of these AMG-projecting neurons in MFC and influence behavior (Marek et al., 2018). Interestingly, our anatomical data revealed that dcHPC and vHPC present significantly distinct projection patterns to MFC, suggesting that their innervation may differentially influence the projection neurons in the MFC subregions. Thus, to confirm the distinct targeting of dcHPC or vHPC inputs to the MFC subregions, we next applied optogenetics and whole-cell recordings of AMG-projecting neurons in MFC in mice (23 male). First, ChR2 was expressed in either dcHPC or vHPC by AAV transduction, and in parallel, either the retrograde AAVrg-hSyn-mCherry or Red Retrobeads were injected in AMG. We then obtained whole-cell voltage–clamp recordings from AMG-projecting neurons in acute coronal slices of MFC while optogenetically stimulating the terminal axons from dcHPC or vHPC (Fig. 2*A*, left). Recorded neurons were labeled with biocytin for later verification of their location within the MFC subregions. We assessed the relative excitatory and inhibitory drive by recording light-evoked synaptic input at holding potential of −70 mV for EPSCs and at +10 mV for IPSCs. Since HPC innervations are known to be of glutamatergic or excitatory nature in MFC (Thierry et al., 2000; Tierney et al., 2004; Parent et al., 2010), we assumed that the IPSC responses would be mediated by disynaptic inhibition from local interneurons (Fig. 2*A*, right).

We found that the connectivity between dcHPC and vHPC with the MFC subregions reflected our anatomical observations. Optical stimulation of dcHPC axon fibers evoked responses in AMG-projecting neurons mainly located in DP (66.7%, 10 out of 15 cells) and MO (35.3%, 6 out of 17 cells), whereas no recorded neuron responded in IL (0 out of 15 cells) and PLv (0 out of 1 cell; Fig. 2*B*). In contrast, the light stimulation of vHPC axon fibers evoked responses in all recorded AMG-projecting neurons in IL (19 out of 19 cells) and PLv (7 out of 7 cells) and to a lesser extent in MO (68.8%, 11 out of 16 cells) and DP (42.9%, 3 out of 7 cells; Fig. 2*C*). The optical stimulation of either dcHPC or vHPC terminals evoked excitatory and inhibitory responses in the AMG-projecting neurons, recorded at −70 mV (EPSC) and at +10 mV (IPSC), respectively (Fig. 2*D*,*E*). The mean latencies of the EPSC and IPSC responses were significantly different, with IPSC lagging EPSC when optically stimulating the vHPC fibers (EPSC, 5.1 ± 0.3 ms; IPSC, 7.3 ± 0.3 ms; mean ± SEM; *p* < 0.0001; two-tailed *t* test) and the dcHPC fibers in MFC (EPSC, 5.6 ± 0.4 ms; IPSC, 8.7 ± 0.7 ms; mean ± SEM; *p* = 0.0003; two-tailed *t* test). Furthermore, in both vHPC and dcHPC cases, IPSCs, but not EPSCs, were abolished when isolating the monosynaptic inputs by perfusing the recording solution with a mix of TTX (1 µM), 4-AP (100 µM), and elevated Ca^2+^ (4 mM). IPSCs were also abolished by applying gabazine (10 µM) to the extracellular solution, which blocks the GABA_A_ receptors (data not shown). These observations indicated that, as proposed above (Fig. 2*A*, right) and in line with the postulated glutamatergic nature of the HPC inputs to MFC (Thierry et al., 2000; Tierney et al., 2004; Parent et al., 2010), the evoked IPSC responses may be indeed a consequence of the HPC innervation of the inhibitory local circuit in MFC. By comparing the ratio of excitatory to inhibitory (E/I) responses from the responsive cells, we observed that inputs from both dcHPC and vHPC seem to drive substantial disynaptic inhibition in AMG-projecting neurons, with no significant differences within the MFC subregions (Fig. 2*F*; *p* = 0.46; Kruskal–Wallis test). Interestingly, when the E/I ratios from the responsive cells in MFC were pooled together and analyzed as a group for each HPC case, vHPC seemed to have an overall stronger excitatory drive over the AMG-projecting neurons in MFC than dcHPC (Fig. 2*G*; vHPC, 0.45; *n* = 40 cells; dcHPC, 0.27; *n* = 16 cells; *p* = 0.03; two-tailed Mann–Whitney test).

These results confirm that dcHPC and vHPC axons contact neurons in different subregions of MFC in accordance with the projection patterns described in our tracing experiments, i.e., dcHPC mainly engages neurons located in DP and MO, whereas vHPC innervates neurons in all MFC subregions, though with a substantial preference for neurons located in IL and PLv. Furthermore, these two parallel pathways presented different overall ratios of excitatory and inhibitory influence on MFC, suggesting that dcHPC and vHPC may be innervating different proportions of excitatory and inhibitory neurons in MFC.

### Distribution of postsynaptic neurons in MFC subregions targeted by dcHPC and vHPC in mice

**Figure 3.**
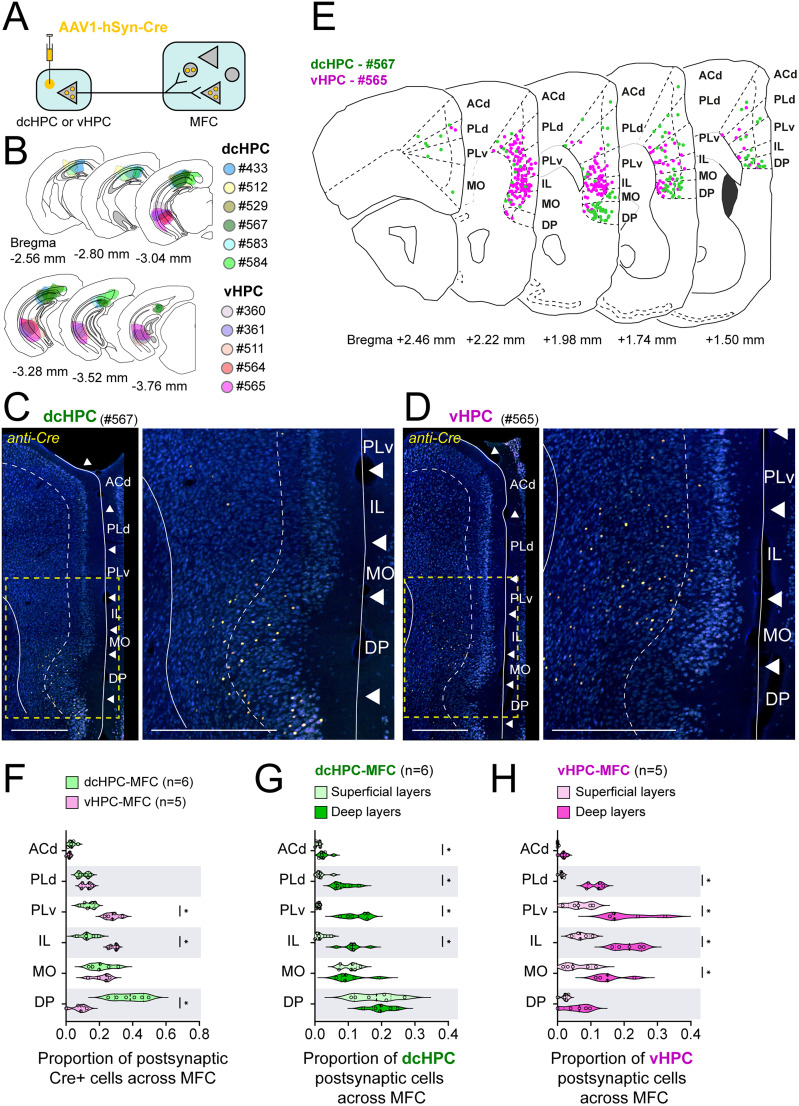
Anterograde transsynaptic spread of AAV1-Cre injections from dcHPC and vHPC target superficial and deep layers of MFC in mice. ***A***, AAV1-hSyn-Cre was injected in either dcHPC or vHPC resulting in expression in HPC projection neurons (data not shown). The transsynaptic spread to postsynaptic neurons was detected by immunostaining against Cre. Note that postsynaptic neurons likely include principal neurons and interneurons in MFC which, in the scheme, are represented by triangles and circles, respectively. The present experiments do not include a further differentiation between interneurons and principal neurons. ***B***, Summary of AAV1-Cre injection sites along HPC displayed in coronal sections. Injection sites in dcHPC and vHPC are shown in cool and warm colors, respectively. ***C***, Representative samples of MFC coronal sections showing the distribution of postsynaptic Cre-expressing neurons in MFC for dcHPC injections (left, case #567) and high-magnification image of the boxed region (right). Scale bars, 500 µm. ***D***, Representative samples of MFC coronal sections showing the distribution of postsynaptic Cre-expressing neurons in MFC for vHPC injections (left, case #565) and high-magnification image of boxed region (right). Scale bars, 500 µm. ***E***, Representative scheme showing the distribution of postsynaptic Cre-labeled neurons across the anterior–posterior axis of MFC for one dcHPC (case #567, green) and one vHPC (case #565, magenta) injection. ***F***, Proportion of the number of Cre-labeled cells in a MFC subregion among the total Cre-labeled neurons across MFC sections for dcHPC (green, *n* = 5) and vHPC (magenta, *n* = 5) injections. Data are presented as violin plots, with each circle corresponding to one sample. Values from all subregions sum up to 1 for one individual sample. Mann–Whitney test corrected for multiple comparisons using the Holm–Šídák method: *p < 0.05. ***G***,***H***, Laminar distribution of postsynaptic Cre-labeled cells in superficial and deep layers of MFC subregions for dcHPC (***E***) and vHPC (***F***) injections. Data are presented as violin plots, with each circle corresponding to one sample. Values from superficial and deep layers of all subregions sum up to 1 for one individual sample. Mann–Whitney test corrected for multiple comparisons using the Holm–Šídák method: *p < 0.05.

**Figure 4.**
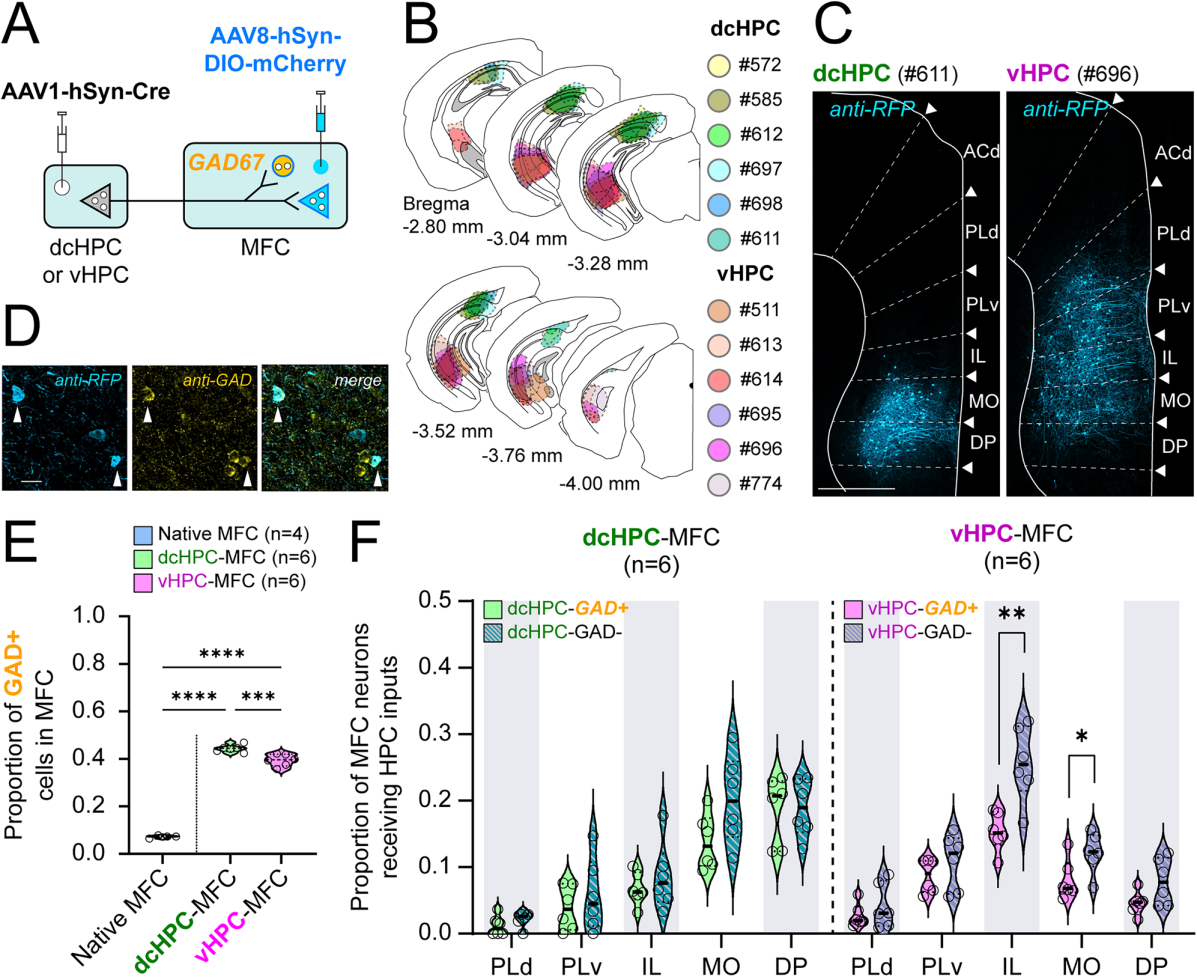
dcHPC and vHPC inputs to MFC innervate GABAergic and non-GABAergic neurons in mice. ***A***, AAV1-hSyn-Cre was injected in either dcHPC or vHPC and was expressed in HPC projection neurons. The transsynaptic spread led to Cre expression in postsynaptic neurons, including principal neurons and interneurons in MFC. In parallel, AAV8-hSyn-DIO-mCherry was injected in MFC leading to Cre-dependent mCherry expression in postsynaptic neurons in MFC. Postsynaptic interneurons were subsequently differentiated by immunostaining against GAD67, as illustrated in ***D–F***. In the scheme, principal neurons and interneurons are represented by triangles and circles, respectively. ***B***, Summary of AAV1-hSyn-Cre injection sites along the HPC displayed in coronal sections. Injection sites in the dcHPC and vHPC are shown in cool and warm colors, respectively. ***C***, Representative samples with AAV1-hSyn-Cre injection in either dcHPC (left, case #611) or vHPC (right, case #696). Images show the postsynaptic Cre-dependent mCherry expression in a coronal section of MFC. Scale bar, 500 µm. ***D***, Representative sample showing the mCherry expression of postsynaptic neurons and colocalization with the GAD67 antibody labeling as used to identify the GABAergic neurons among the HPC-MFC postsynaptic cells. Scale bar, 20 µm. ***E***, Proportion of cells coexpressing mCherry and GAD67 among the total mCherry+ neurons receiving dcHPC (green, *n* = 6) or vHPC (pink, *n* = 6) inputs in MFC. The proportions were compared with the native presence of GAD67 in MFC (light-blue, *n* = 4). One-way ANOVA, followed by post hoc Tukey's multiple-comparison test: ***p < 0.001; ****p < 0.0001. ***F***, Subregional distribution of GABAergic (GAD+) and non-GABAergic (GAD−) neurons in MFC receiving inputs from dcHPC (left, *n* = 6, green tones) and vHPC (right, *n* = 6, pink tones) Cre injections. The proportions of inputs to GAD+ and GAD− cells across MFC subregions sum up to 1 for each injection sample. Data are presented as violin plots, where each circle corresponds to one sample. Two-tailed *t* tests: **p < 0.01; *p < 0.05.

To identify the postsynaptic neurons receiving HPC inputs in MFC, we next used an anterograde transsynaptic tagging strategy with adeno-associated virus serotype 1 (AAV1) in mice (11 male; Fig. 3*A*). When injected at high titer, AAV1 can efficiently spread anterogradely from excitatory projection neurons to both excitatory and inhibitory postsynaptic cell types (Zingg et al., 2017, 2020). Although AAV1 can also transport retrogradely at high titer, this is not of concern in this experiment since these two regions are mainly unidirectionally connected through the direct projections from HPC to MFC (Andrianova et al., 2023). Here, we injected a Cre-expressing AAV1 (AAV1-hSyn-Cre) in dcHPC (*n* = 6) or vHPC (*n* = 5; Fig. 3*B*) and identified the postsynaptic neurons in MFC, either by immunostaining against the Cre-recombinase (Fig. 3) or by the presence of fluorescent protein expressed via a Cre-dependent AAV injected in MFC (Figs. 4, 6). In line with the observations of our anterograde tracing experiments (Fig. 1), dcHPC innervated mainly neurons in DP and MO (Fig. 3*C*,*E*), whereas vHPC innervated mainly neurons in IL, PLv, and MO (Fig. 3*D*,*E*). To describe the distribution of the neurons receiving HPC inputs across the MFC subregions, we quantified a subregional proportion as the number of Cre-labeled cells in a subregion among the total number of Cre-labeled cells in MFC. The distribution of HPC postsynaptic cells across the MFC subregions (Fig. 3*F*) presented comparable patterns to the ones observed in our previous experiment using conventional anterograde tracing (Fig. 1*C*). Compared with dcHPC, projections from vHPC innervated a larger proportion of neurons in IL and PLv (dcHPC vs vHPC; IL, *p* = 0.03; PLv, *p* = 0.03; Mann–Whitney test). In contrast, dcHPC presented a larger proportion of inputs into DP than vHPC (dcHPC vs vHPC; *p* = 0.03; Mann–Whitney test). The proportion of cells receiving inputs from dcHPC or vHPC were similar in ACd, PLd, and MO (dcHPC vs vHPC; ACd, *p* = 0.2; PLd, *p* = 0.4; MO, *p* > 0.99; Mann–Whitney test). Although we did not look into the distribution of Cre-labeled cells along the anterior–posterior axis of MFC, it seems that whereas vHPC targets IL and PLv densely in middle sections of MFC, dcHPC projections seem denser in DP in middle-to-posterior sections of MFC (Fig. 3*E*).

We also noticed that the distribution of Cre-expressing cells differed across layers. In general, deep layers (L5/6) of MFC presented larger proportions of Cre-labeled cells than superficial layers (L1/2/3) of MFC. The proportion of neurons receiving inputs from dcHPC (Fig. 3*G*) was larger in deep layers than in superficial layers in ACd, PLd, PLv, and IL (deep vs sup; ACd, *p* = 0.04; PLd, *p* = 0.01; PLv, *p* = 0.01; IL, *p* = 0.01; Mann–Whitney test). In MO and DP, however, Cre-labeled cells were similarly distributed across superficial and deep layers (deep vs sup; MO, *p* = 0.9; DP, *p* = 0.9; Mann–Whitney test). Regarding the proportion of labeled neurons receiving inputs from vHPC (Fig. 3*H*), we observed that this was larger in deep layers than superficial layers in MO, IL, PLv, and PLd (deep vs sup; MO, *p* = 0.047; IL, *p* = 0.047; PLv, *p* = 0.047; PLd, *p* = 0.047; Mann–Whitney test), whereas the proportions of labeled neurons for deep and superficial layers in ACd and DP were similar (deep vs sup; ACd, *p* = 0.09; DP, *p* = 0.1; Mann–Whitney test). These observations confirm that dcHPC and vHPC project differently to MFC; dcHPC mainly innervates neurons in superficial and deep layers of DP, whereas vHPC sends mainly inputs to deep layers of IL and PLv.

### dcHPC and vHPC innervate significant proportions of GABAergic neurons in the mouse MFC

Previous studies have reported that projections from vHPC to MFC play a crucial role in regulating memory and emotional-related behavior through the recruitment of GABAergic neurons in MFC (Padilla-Coreano et al., 2016; Marek et al., 2018; Phillips et al., 2019; Anastasiades and Carter, 2021). Interestingly, the results from our whole-cell recordings suggested that the overall E/I ratio in MFC tends to be more inhibitory upon optogenetic stimulation of axon fibers originating in dcHPC than in vHPC (Fig. 2*G*). To verify whether dcHPC and vHPC innervate different proportions of GABAergic and non-GABAergic neurons in MFC, we used the anterograde transsynaptic tagging method to label the neurons receiving HPC inputs in MFC (12 males): AAV1-hSyn-Cre was injected into either dcHPC (*n* = 6) or vHPC (*n* = 6), and in parallel, a Cre-dependent AAV expressing mCherry (AAV8-hSyn-DIO-mCherry) was injected into MFC (Fig. 4*A,B*). Consistent with the labeling patterns observed in the above sections, for dcHPC injections, mCherry+ cells were localized mainly in DP and MO (Fig. 4*C*, left), whereas for vHPC injections, mCherry+ cells were spread along MO, IL, and PLv subregions (Fig. 4*C*, right).

Next, by immunostaining against glutamic acid decarboxylase (GAD67), the mCherry+ cells were differentiated as GABAergic (GAD+) or non-GABAergic (GAD−) neurons (Fig. 4*A*,*D*). The proportions of GAD+ neurons among the total of mCherry+ cells in MFC receiving inputs from dcHPC or vHPC were calculated and then compared with the native proportion of GAD+ cells in MFC, corresponding to the overall proportion of GABAergic neurons present in MFC (Fig. 4*E*). Both dcHPC and vHPC projections innervated a significant large proportion of GAD+ cells considering the native presence of GAD+ cells in MFC (native MFC, 0.072 ± 0.006; dcHPC-MFC, 0.44 ± 0.01; vHPC-MFC, 0.39 ± 0.02; mean ± SD; *F*_(2,13)_ = 543.3; *p* < 0.0001; one-way ANOVA; dcHPC-MFC vs native MFC, *p* < 0.0001; vHPC-MFC vs native MFC, *p* < 0.0001; one-way ANOVA followed by post hoc Tukey's multiple-comparison test). Interestingly, a larger proportion of dcHPC postsynaptic neurons in MFC were GAD+ than among vHPC postsynaptic neurons (dcHPC-MFC vs vHPC-MFC, *p* = 0.0005; one-way ANOVA followed by post hoc Tukey's multiple-comparison test).

To further characterize the dcHPC-MFC and vHPC-MFC circuits, we calculated the proportions of GAD+ and GAD− neurons among the total mCherry+ cells receiving HPC inputs for each MFC subregion (Fig. 4*F*). Because mCherry expression was absent in ACd, this subregion was excluded from this analysis. Projections from dcHPC innervated similar proportions of GAD+ and GAD− cells in the MFC subregions (GAD+ vs GAD−; PLd, *p* = 0.21; PLv, *p* = 0.48; IL, *p* = 0.44; MO, *p* = 0.07; DP, *p* = 0.95; two-tailed *t* test). In contrast, vHPC innervated similar proportions of GAD+ and GAD− cells in MFC subregions with the exception of IL and MO (GAD+ vs GAD−; PLd, *p* = 0.38; PLv, *p* = 0.29; DP, *p* = 0.07; two-tailed *t* test). In IL and MO, a larger proportion of GAD− than GAD+ neurons received vHPC inputs (IL, *p* = 0.003; MO, *p* = 0.04; two-tailed *t* test), which suggested that vHPC favors non-GABAergic innervation in IL and MO.

**Figure 5.**
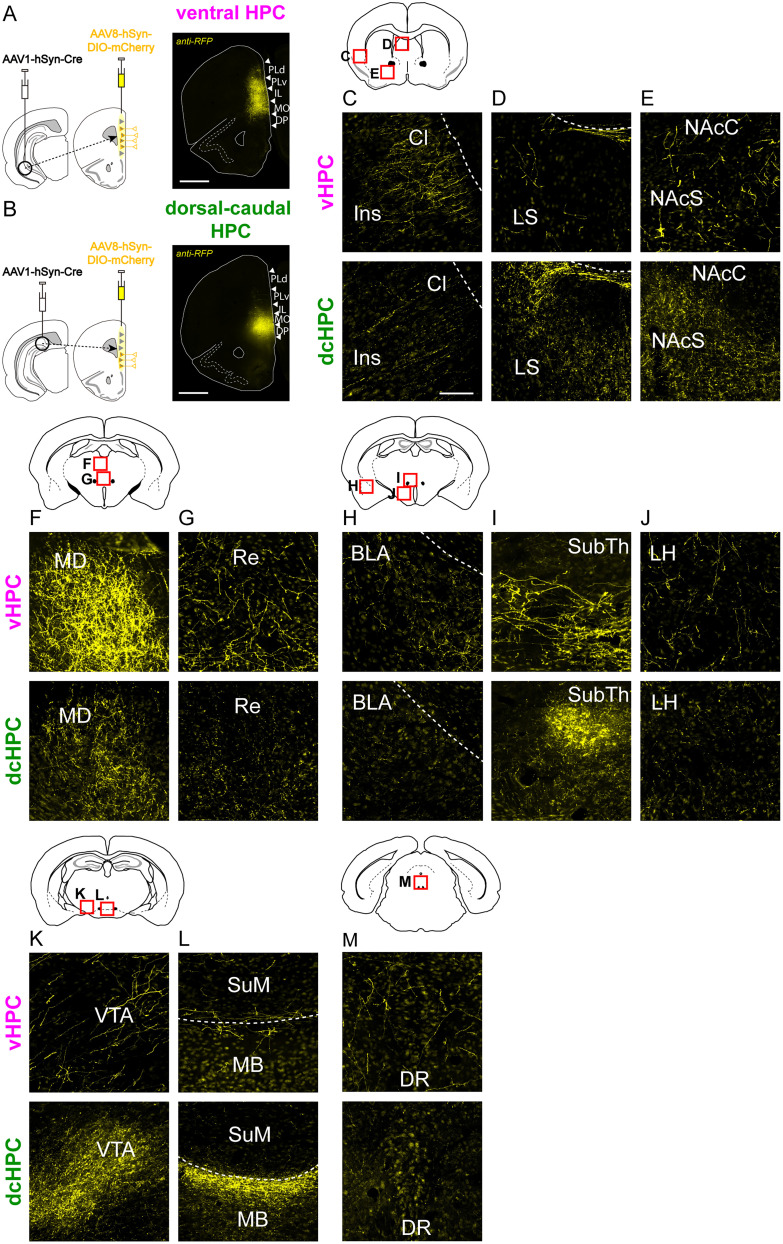
Brain regions targeted by MFC neurons receiving inputs from dcHPC or vHPC in mice. ***A***,***B***, Representative image showing mCherry-labeled neurons in MFC receiving inputs from vHPC (***A***) or dcHPC (***B***). Note that vHPC postsynaptic neurons are mainly distributed across MO, IL, and PLv subregions, whereas dcHPC postsynaptic neurons are mainly distributed across DP and MO subregions in MFC (white arrow heads indicate the borders between subdivisions). Scale bars, 1,000 µm. ***C–M***, The presence of mCherry+ terminals from MFC neurons innervated by vHPC (top) or by dcHPC (bottom) in the claustrum (Cl) and insular cortex (Ins, ***C***), lateral septum (LS, ***D***), nucleus accumbens core and shell (NAcC, NAcS, ***E***), mediodorsal thalamus (MD, ***F***), nucleus Re (***G***), basolateral AMG (BLA, ***H***), submedial thalamic nucleus (SubTh, ***I***), lateral hypothalamus (LH, ***J***), ventral tegmental area (VTA, ***K***), mammillary and supramammillary body (MB, SuM, ***L***), and dorsal raphe (DR, ***M***). Scale bar, 100 µm.

A visual inspection of the mCherry+ neuron axon terminals throughout the brain (Fig. 5) showed that MFC neurons receiving inputs from vHPC and dcHPC (Fig. 5*A*,*B*) target the claustrum, insular cortex, septum, nucleus accumbens, mediodorsal thalamus, basal AMG, hypothalamus, ventral tegmental area, and dorsal raphe nucleus (Fig. 5*C*–*M*). These observed projections are in line with previous anatomical studies on the efferent projections from MFC (Hurley et al., 1991; Vertes, 2004; Gabbott et al., 2005). Interestingly, MFC neurons receiving dcHPC inputs targeted distinctively the mammillary body (Fig. 5*L*), ventral tegmental area (Fig. 5*K*), and submedial nucleus of the thalamus (Fig. 5*I*), consistent with projections previously ascribed to DP (Hurley et al., 1991; Geisler and Zahm, 2005; Mathiasen et al., 2019).

These results altogether show that both dcHPC and vHPC innervate significant proportions of GABAergic cells in the MFC subregions, and furthermore, they suggest that dcHPC and vHPC may exert different excitatory/inhibitory influence onto diverse projection neurons in MFC.

### dcHPC and vHPC recruit parvalbumin- and somatostatin-expressing cells in the mouse MFC

Our anatomical observations led to the conclusion that GABAergic neurons are among the monosynaptically innervated neurons in MFC. Previous reports are in line with this and further suggest that vHPC projections may regulate the network dynamics in MFC through the recruitment of different types of inhibitory interneurons found in MFC, including parvalbumin- (PV+), somatostatin- (SOM+), vasoactive intestinal peptide- (VIP+), and cholecystokinin (CCK+)-expressing neurons (Tremblay et al., 2016; Ährlund-Richter et al., 2019; Anastasiades and Carter, 2021).

**Figure 6.**
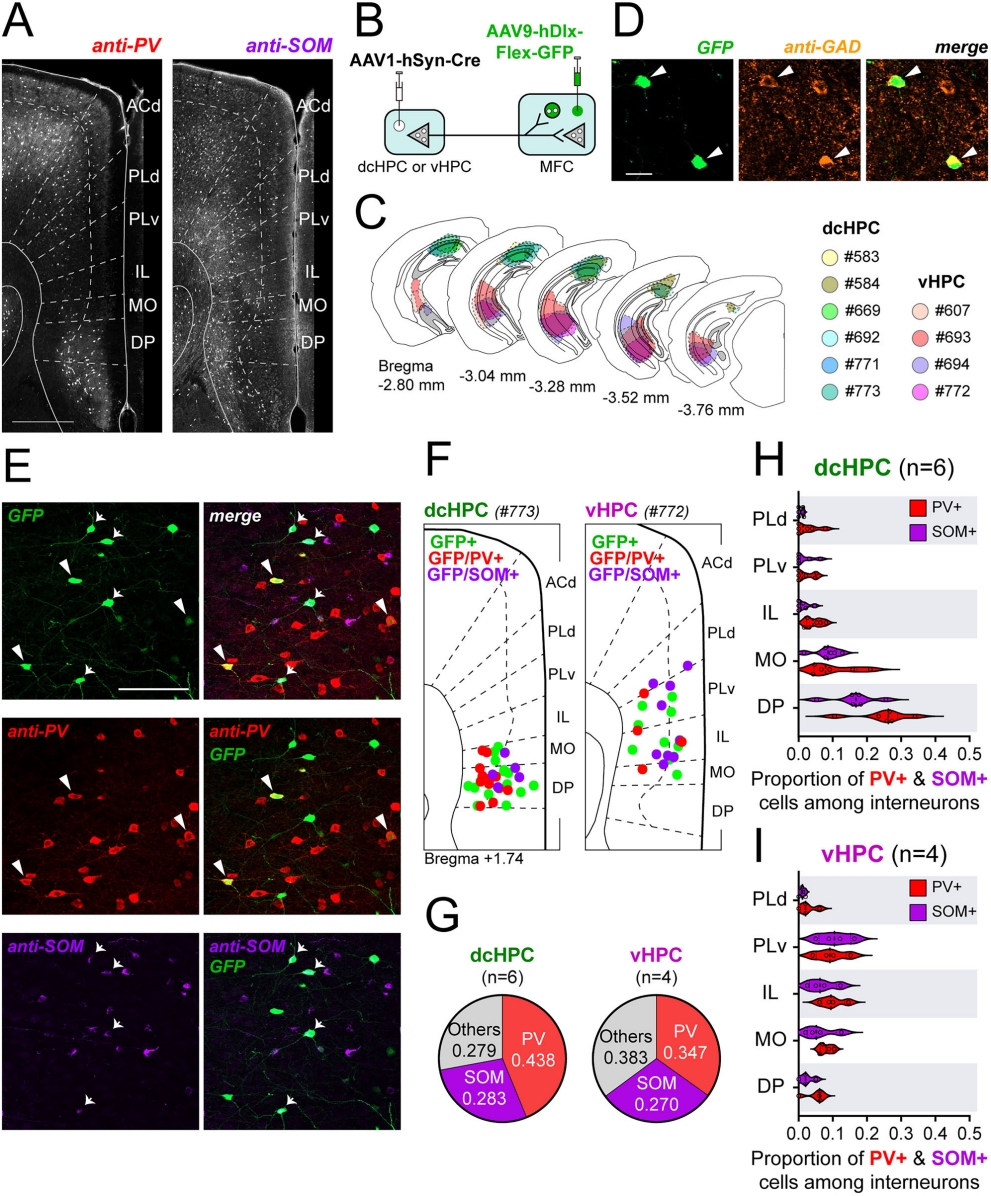
dcHPC and vHPC innervate both PV and SOM interneurons in the mouse MFC. ***A***, Representative images showing the native distribution of the indicated marker expression (left, PV; right, SOM) in the subregions of MFC. Scale bar, 500 µm. ***B***, AAV1-hSyn-Cre was injected in either dcHPC or vHPC and expressed in HPC projection neurons. The transsynaptic spread led to Cre expression in postsynaptic neurons, including principal neurons and interneurons in MFC. In parallel, AAV9-hDlx-Flex-GFP was injected in MFC, leading to Cre-dependent GFP expression in postsynaptic interneurons in MFC. In the scheme, principal neurons and interneurons are represented by triangles and circles, respectively. ***C***, Summary of AAV1-hSyn-Cre injection sites along HPC in coronal sections. Injection sites in dcHPC and vHPC are shown in cool and warm colors, respectively. ***D***, Micrographs showing the viral GFP expression of labeled GABAergic neurons and colocalization with the GAD67 antibody. Scale bar, 20 µm. ***E***, Micrographs showing the viral GFP expression and colocalization of GFP+ cells and the indicated markers (PV+ in red, SOM+ in violet). Scale bar, 100 µm. ***F***, Representative schemes showing the distribution of the viral GFP-expressing interneurons in MFC receiving inputs from dcHPC (left, case #773) and vHPC (right, case #772) Cre injections that coexpress either PV or SOM across the MFC subregions. ***G***, Pie charts showing the proportion of GFP+ cells coexpressing PV (red) or SOM (violet) among the total GFP-labeled interneurons receiving dcHPC (left, *n* = 6) or vHPC (right, *n* = 4) inputs. ***H***,***I***, Subregional distribution of the GFP+ interneurons receiving HPC inputs in MFC that coexpress the indicated markers PV (red) or SOM (violet) for dcHPC (***H***, *n* = 6) and vHPC (***I***, *n* = 4). Data are presented as violin plots, with each circle corresponding to one sample. For one sample, proportions of PV+ and SOM+ cells across the MFC subregions do not sum up to 1, with the residual proportion corresponding to unidentified GFP+ interneurons. A Mann–Whitney test corrected for multiple comparisons using the Holm–Šídák method.

Two of the main populations of GABAergic neurons in MFC are PV+ and SOM+ interneurons, which have characteristic physiological and morphological properties, and display particular lamination patterns in MFC (Anastasiades and Carter, 2021). Fast-spiking PV+ interneurons target cell bodies and proximal dendrites of pyramidal neurons leading to a high level of feedforward and feedback inhibition (Hu et al., 2014). In contrast, SOM+ interneurons form inhibitory synapses on distal dendritic branches of principal neuron enhancing the selectivity of excitatory inputs (Markram et al., 2004; Gentet et al., 2012) and leading to long-lasting and delayed inhibition (Yang et al., 2021). SOM+ neurons also innervate nearby PV+ cells, thus decreasing PV-mediated inhibition (Pfeffer et al., 2013). Interestingly, immunostained sections of MFC against PV and SOM showed that PV+ neurons are densely localized in the most dorsal and most ventral parts of MFC (Fig. 6*A*, left), whereas SOM+ interneurons seemed to be uniformly distributed across the MFC subregions (Fig. 6*A*, right). We thus hypothesized that dcHPC and vHPC innervate different interneuron types in MFC. To examine this, we used an anterograde transsynaptic tagging strategy (Fig. 6*B*) in which the AAV1-hSyn-Cre was injected in either dcHPC (*n* = 6) or vHPC (*n* = 4; Fig. 6*C*), and in parallel the AAV9-hDlx-Flex-GFP was injected in MFC (10 males). The latter viral vector expresses a Cre-dependent GFP reporter under the control of the hDlx promoter to specifically tag GABAergic neurons as previously reported (Dimidschstein et al., 2016), which we confirmed by immunostaining against GAD67 (Fig. 6*D*). Subsequently, the GFP-expressing neurons were characterized by immunostaining against the markers PV and SOM (Fig. 6*E*). We then calculated the proportions of PV+ and SOM+ interneurons among the total GFP-labeled interneurons in MFC. Our results showed that dcHPC and vHPC innervated different proportions of PV+, SOM+, and other kinds of interneurons in MFC (Fig. 6*F*,*G*). Among the interneurons receiving dcHPC inputs, the PV+ proportion was larger compared with the SOM+ and other non-PV/SOM interneurons (PV+ vs SOM+, *p* = 0.024; PV+ vs others, *p* = 0.021; SOM+ vs others, *p* = 0.997; one-way ANOVA followed by Tukey's multiple-comparison test). In contrast, among the interneurons receiving vHPC inputs, the proportions of PV+ and SOM+ were not significantly different (PV+ vs SOM+, *p* = 0.079; PV+ vs others, *p* = 0.484; SOM+ vs others, *p* = 0.012; one-way ANOVA followed by Tukey's multiple-comparison test). Furthermore, we analyzed the subregional distribution of PV+ and SOM+ interneurons receiving HPC inputs in MFC (Fig. 6*H*,*I*). Among the interneurons receiving dcHPC inputs, PV+ and SOM+ cells were mainly located in DP and MO (Fig. 6*H*). In contrast, PV+ and SOM+ cells receiving vHPC inputs were mainly located in PLv and IL (Fig. 6*I*). Note, however, that we did not find any significant differences in the proportions of postsynaptic PV+ and SOM+ cells within any MFC subregion for either dcHPC (Fig. 6*H*) or vHPC (Fig. 6*I*) cases.

These results show that although dcHPC and vHPC project to different subregions in MFC, they innervate similar proportions of PV+ and SOM+ interneurons in the target subregions. However, in contrast to vHPC projections, dcHPC presented a trend to recruit a larger overall proportion of PV+ cells over other types of interneurons in MFC, which may implicate an increased perisomatic inhibition of MFC neurons by dcHPC innervation, a possibility that still needs to be tested.

### dcHPC and vHPC project differently to MFC subregions in rats

**Figure 7.**
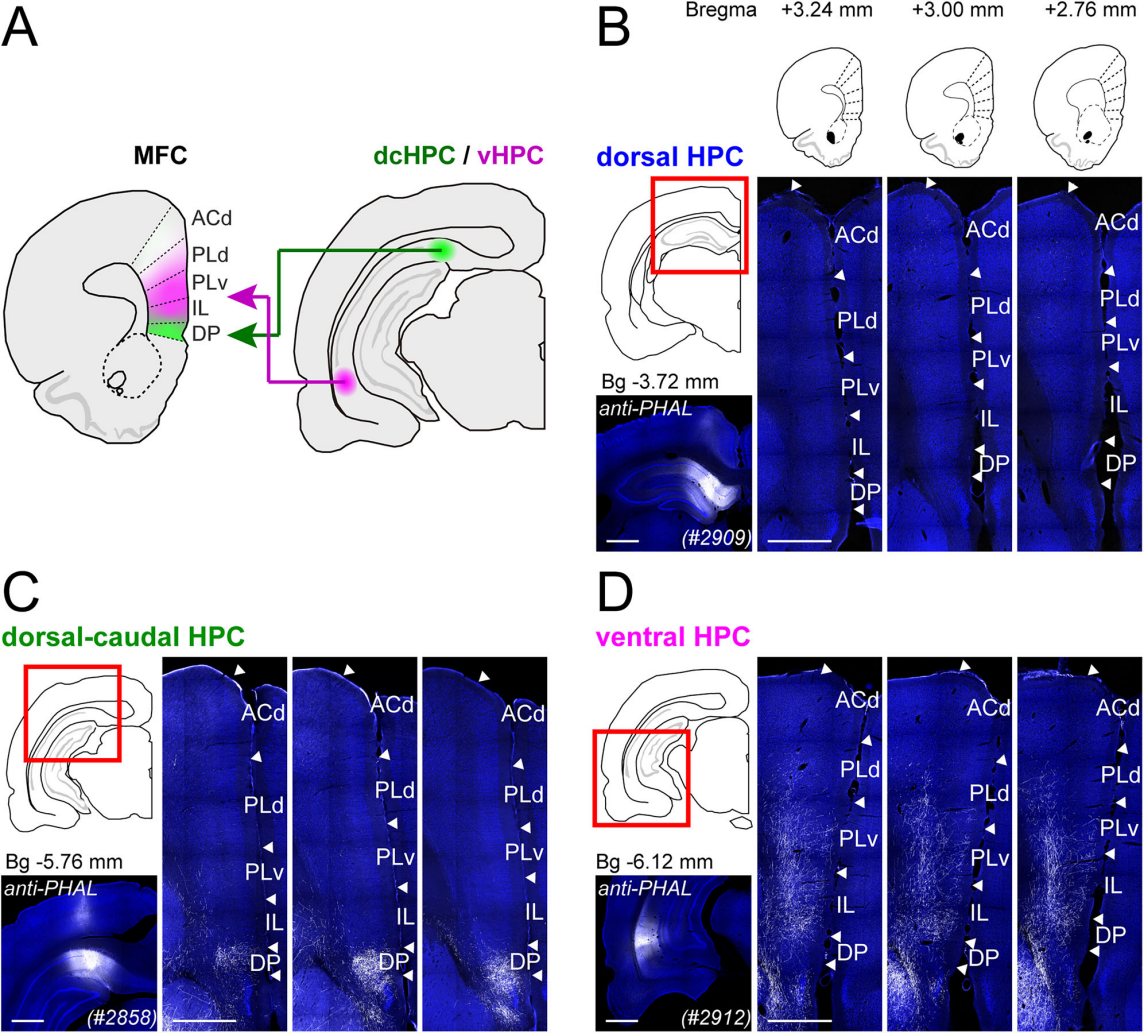
Anterogradely labeled fibers originating along the dorsoventral axis of HPC are differently distributed across MFC subregions in rats. ***A***, Summary illustration of parallel pathways from dcHPC and vHPC to MFC subregions. ***B–D***, Representative samples of anterogradely labeled fibers in MFC (at three coronal levels as shown in the top inset) for injections in the most dHPC (#2909-PHAL, ***B***), dcHPC (#2858-PHAL, ***C***), and vHPC (#2912-PHAL, ***D***). Scale bars, 1 mm.

The differences in projection patterns between the dcHPC-MFC and vHPC-MFC circuits shown above have not been clearly addressed in previous studies. One possible explanation is species differences. While previous studies used rats (Jay and Witter, 1991; Cenquizca and Swanson, 2007), our experiments used mice. To assess the consistency of the HPC-MFC circuits across rodent species, we performed anterograde tracing experiments in rats (three males; Fig. 7). Similar to the projection patterns in mice (Fig. 7*A*), after tracer injections in dHPC, labeling in MFC was weak to absent (Fig. 7*B*). Injections in dcHPC resulted in very dense fiber labeling in DP, the most vMFC subregion (Fig. 7*C*). Conversely, injections in vHPC resulted in dense labeled fibers in IL and PLv subregions (Fig. 7*D*). These data thus indicate that the projection patterns of HPC-MFC pathways are similar between mice and rats. It is important to notice, however, that although the MFC is similar in rats and mice, there are subregional delineation differences, i.e., the presence of MO in posterior portions of MFC in mice, but restricted to rostral portions of MFC in rats.

### Multisynaptic projections from MFC to dcHPC and vHPC are organized along the dorsoventral axis in rats

Surprisingly, the topographical patterns of the direct HPC-MFC projection differed significantly from the known projection patterns of the reverse pathway from MFC to HPC. Previous anatomical studies have established that the direct MFC-HPC projections are apparently absent and are instead mediated indirectly via either midline thalamic nuclei or the entorhinal cortex (EC; Vertes et al., 2007; Agster and Burwell, 2013; Varela et al., 2014). A previous retrograde tracing study using pseudorabies virus demonstrated that these indirect pathways are organized topographically along the dorsoventral axis of HPC: dMFC projects to dHPC through dorsolateral EC or the anterior thalamus, while vMFC projects to vHPC through caudomedial EC or the midline thalamus (Prasad and Chudasama, 2013).

**Figure 8.**
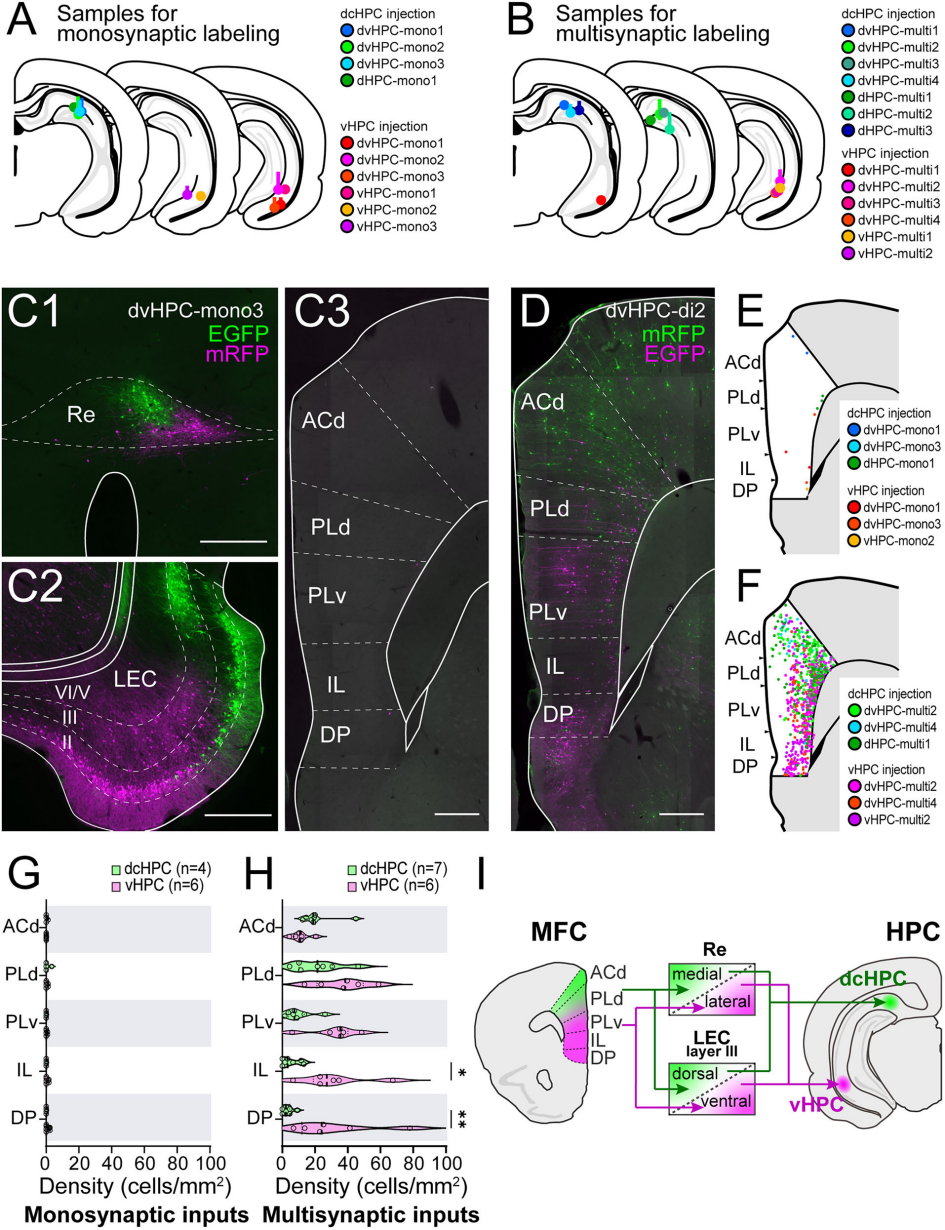
Organization of multisynaptic inputs from MFC to HPC along the dorsoventral axis in rats. ***A***,***B***, Summary of injection sites of RV vectors in the rat dcHPC and vHPC (16 males). Each injection is illustrated with a different color. Either a glycoprotein-deleted RV vector or a propagation-competent RV vector with short survival time was used to examine monosynaptic inputs to dcHPC (*n* = 4) and vHPC (*n* = 6) (***A***). A propagation-competent RV vector with longer survival time was used to examine disynaptic inputs to dcHPC (*n* = 7) and vHPC (*n* = 6) (***B***). ***C***, Fluorescence micrograph of retrograde labeling in Re (***C*1**), LEC (***C*2**), and MFC (***C*3**) of a monosynaptically labeled sample (case dvHPC-mono3). Neurons infected by the RV vector injected to dcHPC are shown in green, while neurons infected by the vHPC-injected vector are shown in magenta. Scale bars, 500 µm. ***D***, Fluorescence micrograph of retrograde labeling in MFC of a multisynaptically labeled sample (case dvHPC-multi2). Scale bar, 500 µm. ***E***,***F***, Distribution of labeled MFC neurons from three representative samples are overlaid for monosynaptic (***E***) and multisynaptic samples (***F***) for injections in dcHPC (cool colors) and vHPC (warm colors). ***G***,***H***, Density of labeled neurons across MFC subregions is shown for monosyanptic- (***G***) and multisynaptic-labeling samples (***H***) after dcHPC (green) and vHPC (pink) injections. Each circle represents the individual density of labeled cells in the subregion for one injection sample. Data are presented as violin plots. Mann–Whitney test corrected for multiple comparisons using the Holm–Šídák method: **p < 0.01; *p < 0.05. ***I***, Summary illustration of parallel disynaptic HPC pathways from dMFC and vMFC to dcHPC and vHPC, via Re or LEC L3 neurons.

To compare the projection patterns of the direct HPC-MFC pathway with those of the indirect MFC-HPC pathway, we reexamined the organization of multisynaptic MFC inputs to the dcHPC and vHPC by reanalyzing samples obtained from previous retrograde transsynaptic tracing experiments with RV in rats, some of which have been reported previously (Ohara et al., 2013). We first examined the brain regions with direct projections to HPC (seven males) by using either a glycoprotein-deleted RV vector (ΔG −RV), which can be used as a nontranssynaptic retrograde tracer (Ohara et al., 2013), or a propagation-competent RV vector, labeling first-order projection neurons with 2 d of survival time (Ohara et al., 2009; Fig. 8*A*). Irrespective of the RV vector used, many retrogradely labeled neurons were observed in the thalamic nucleus reuniens (Re) and L2 and 3 of EC with topographical labeling patterns depending on the position of the injection site along the HPC longitudinal axis (Fig. 8*C*1,2). The RV vector injected into dcHPC (*n* = 4) resulted in labeled neurons positioned medially in Re and dorsally in lateral EC (LEC), whereas the neurons labeled by the RV vector injected into vHPC (*n* = 6) were preferentially found laterally in Re and ventrally in LEC. In all of the cases, only very few labeled neurons were observed in MFC, which corroborates previous studies that MFC does not, or only very sparsely, projects directly to HPC (Hurley et al., 1991; Takagishi and Chiba, 1991; Buchanan et al., 1994; Fig. 8*C*3,*E*,*G*).

To examine the disynaptic inputs to HPC, we next used the propagation-competent RV vector with 4–5 d of survival time, which is the optimal survival time for the RV vector to transsynaptically label the second-order projection neurons (nine males; Fig. 8*B*). However, since the propagation speed of RV is influenced by the synaptic strength and axonal length (Ohara et al., 2009), there is a possibility that interneurons with short axons may be infected more rapidly than projection neurons, and thus, some transsynaptically labeled neurons might not be true second-order neurons but rather third-order neurons labeled via local circuits. Therefore, in the following section, we refer to these inputs as multisynaptic rather than strictly disynaptic.

In these samples, the number of labeled neurons dramatically increased in MFC, and the two populations of labeled cells exhibited a topographical distribution (Fig. 8*D*,*F*,*H*). The neurons labeled by the ventral injection (*n* = 6) were prominent in the ventral part of MFC, including DP, IL, and PLv. In contrast, neurons labeled by the dorsal–caudal injection (*n* = 7) were mainly distributed in the dorsal part of MFC, particularly in ACd. The density of labeled neurons in PLd was similar for dorsal and ventral injections. Interestingly, we did not see such massive MFC labeling when the RV was injected specifically in DG (Ohara et al., 2013), despite a large number of first-order–labeled cells that were observed in L2 of LEC. Thus, the combined experimental data of this series of tracing experiments indicate that MFC neurons disynaptically project to CA1/SUB either via Re or L3 of EC in a topographical manner along the dorsoventral axis (Fig. 8*I*). The overall topographical organization of these multisynaptic MFC-HPC pathways presented here is in line with the one described previously (Prasad and Chudasama, 2013), though our results did not corroborate the presence of the additional relay in the anterior thalamic nuclei for the dorsal HPC pathways.

Taken together, these rat anatomical data indicate that there is a cross talk between the indirect MFC-HPC circuit and the direct HPC-MFC circuit. The information from MFC to ventral CA1/SUB, originated in PLd, PLv, IL, and DP, will be sent back mainly to PLv and IL. In contrast, information from MFC to dorsal CA1/SUB, originated mainly in ACd and PLd, will be sent back to DP, the most vMFC subregion.

## Discussion

**Figure 9.**
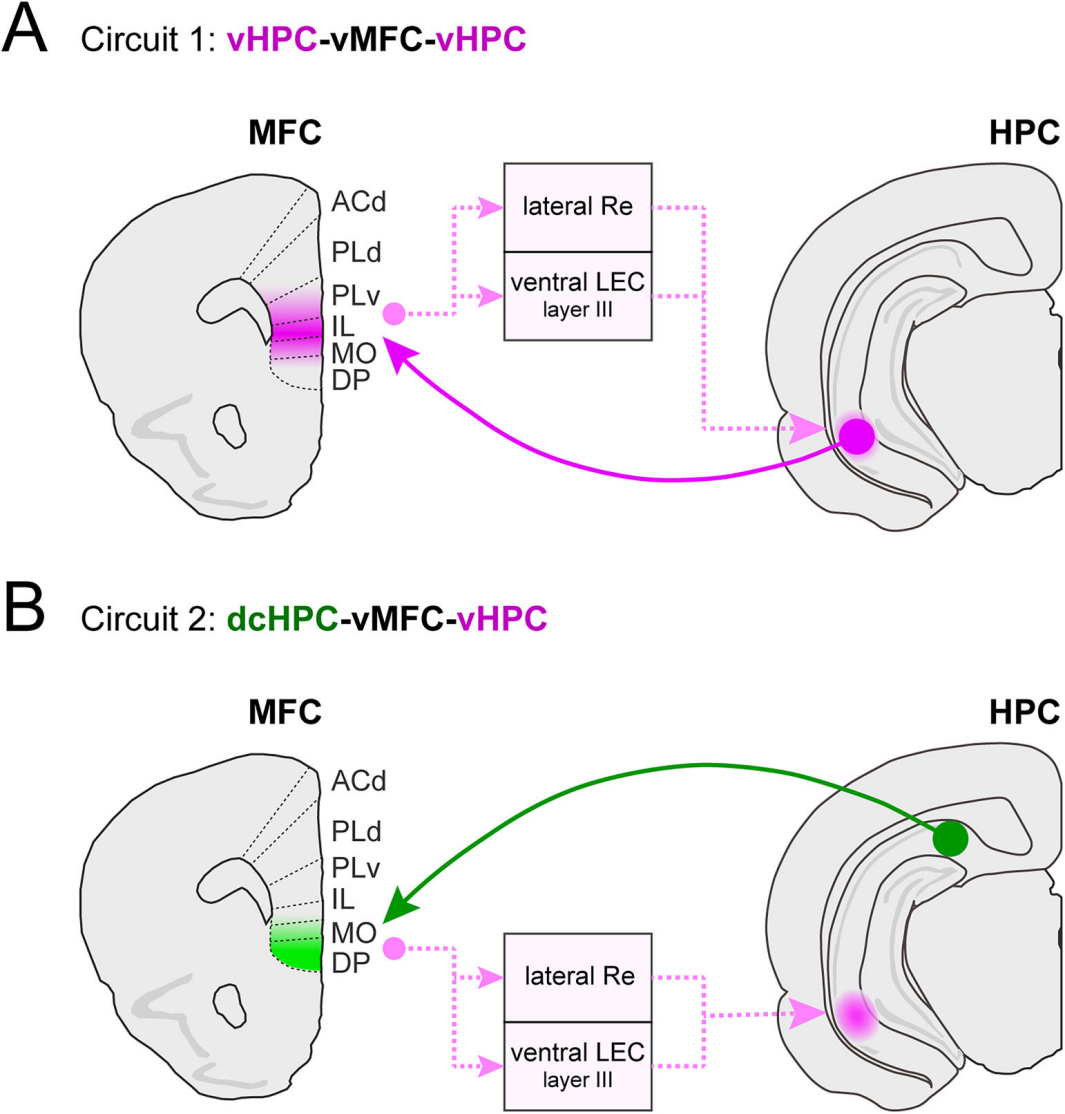
Summary of the connectivity between HPC and the subregions in MFC. ***A***, Reciprocal ventral circuit with a direct projection from vHPC to IL, PLv and MO in vMFC, and an indirect pathway from vMFC back to vHPC through the lateral part of Re and the ventral part of LEC L3 neurons. ***B***, Proposed circuit connecting dcHPC-vMFC-vHPC, with a direct projection from dcHPC to DP and MO in vMFC, and an indirect pathway from vMFC to vHPC through the lateral part of Re and the ventral part of LEC L3 neurons. There is a reciprocal dorsal circuit by which PLd and ACd in dMFC receive limited projections from dcHPC but sends projections back to dcHPC through the medial part of Re and the dorsal part of LEC L3 neurons.

Strong evidence supports the importance of the direct HPC-MFC projection for memory and emotion (Godsil et al., 2013; Spellman et al., 2015; Padilla-Coreano et al., 2016; Sigurdsson and Duvarci, 2016; Wallis et al., 2019; Wang et al., 2021). Most studies focused on the dense vHPC projections, whereas dcHPC projections have been relatively disregarded. Our findings systematically document the HPC-MFC projections along the entire HPC axis, emphasizing the additional relevance of dcHPC-MFC. Notably, our data suggest that vHPC and dcHPC form distinct parallel HPC-MFC pathways, with vHPC mainly targeting IL, PLv, and MO cortices (Fig. 9*A*) and dcHPC mainly targeting DP and MO cortices (Fig. 9*B*). Whole-cell recordings combined with optogenetic stimulation revealed differences in E/I balance between these pathways, which were further confirmed anatomically. Specifically, dcHPC recruits a higher proportion of GABAergic neurons and preferentially targets PV+ interneurons compared with vHPC.

### Organization of the direct HPC-MFC pathways along the dorsoventral axis

By focusing on dcHPC, referring to the dorsal level of distal CA1 and proximal SUB, we revealed a topographical projection pattern from HPC to MFC which was not clearly described in previous anatomical studies in rats (Jay and Witter, 1991; Cenquizca and Swanson, 2007). A study using retrograde tracers showed that PL-projecting neurons were mainly concentrated in vHPC, while IL-projecting neurons spanned the dorsoventral HPC axis in rats (Hoover and Vertes, 2007), though the labeling observed in dcHPC may have resulted from tracer spread to DP. Furthermore, our observations align with those of Bienkowski et al. (2018), who reported that the dorsal–proximal part of SUB, included in dcHPC in our study, targeted mainly DP, whereas the ventral SUB projected densely to IL in mice.

Interestingly, there is evidence that most MFC-projecting CA1 neurons have collaterals to different brain regions (Kim and Cho, 2017; Gergues et al., 2020). These long-range axon collaterals may promote synchronized neural activity and long-term synaptic plasticity across brain areas, potentially influencing different behavioral responses (Kim and Cho, 2017; Gergues et al., 2020).

### HPC activation of inhibitory circuits in MFC

Clinical studies as well as animal models suggest that alterations of cortical E/I balance can give rise to social and cognitive deficits (Yizhar et al., 2011; Nelson and Valakh, 2015; Selimbeyoglu et al., 2017). Several reports demonstrated that vHPC engages distinct interneuron populations in MFC to regulate the network dynamics and influence behavior (Gabbott et al., 2002; Marek et al., 2018; Phillips et al., 2019; Liu et al., 2020). Our whole-cell recordings revealed that dcHPC terminal axons in MFC could also regulate MFC network dynamics, though with a more inhibitory E/I ratio than vHPC. This difference likely results from the fact that dcHPC innervates a higher proportion of GABAergic neurons in MFC than vHPC. Here we focused on PV+ and SOM+ neurons due to their distinct laminar distribution, but VIP+ and CCK+ neurons, known to receive inputs from vHPC (Ährlund-Richter et al., 2019), also warrant investigation.

Adding to the complexity of these inhibitory networks, reports indicate that superficial and deep layers of CA1 differ in preferred inhibitory neuron innervation (Ährlund-Richter et al., 2019; Sun et al., 2019; Sánchez-Bellot et al., 2022). Moreover, the distinct circuits involving the MFC interneurons appear to influence behavior differently. For example, PV+ interneurons are linked to avoidance behavior (Ho et al., 2024), with vHPC innervation of PV+ interneurons in MFC promoting social memory formation (Sun et al., 2020) and inhibiting conditioned fear relapse (Marek et al., 2018). In contrast, SOM+ interneurons are associated with exploration and approach behavior (Soumier and Sibille, 2014; Sánchez-Bellot et al., 2022), and vHPC innervation of SOM+ interneurons facilitates spatial working memory (Abbas et al., 2018).

### Reciprocal circuits connecting HPC and MFC along the dorsoventral axis

In addition to the direct HPC-MFC pathways, we also identified the indirect pathways from MFC back to HPC. We interpret the results of the retrograde transsynaptic tracing as to indicate that these pathways disynaptically connect MFC to HPC and that they are topographically organized along the dorsoventral axis. The dMFC, including ACd and PLd, disynaptically projects to dcHPC, whereas the vMFC subregions PLv and IL disynaptically project to vHPC, consistent with previous report (Prasad and Chudasama, 2013).

Combining the direct HPC-MFC pathways and the indirect MFC-HPC pathways, our findings point to a dorsoventral organization of the bidirectional connectivity between HPC and MFC. We propose a reciprocal ventral circuit where vMFC projects disynaptically to vHPC and receives monosynaptic inputs from vHPC (Fig. 9*A*). Conversely, dMFC projects disynaptically to dcHPC, and, although direct projections from dcHPC to dMFC are limited, dcHPC may reach dMFC disynaptically via LEC (Ohara et al., 2018, 2021, 2023) or thalamic Re (McKenna and Vertes, 2004; Scheel et al., 2020). The bidirectional ventral circuit (Fig. 9*A*) allows vMFC and vHPC to coordinate their activities and the reported shared involvement in emotional, autonomic, and sympathetic functions (Heidbreder and Groenewegen, 2003; Strange et al., 2014), including social behavior (Sun et al., 2020), social memory (Phillips et al., 2019), context-dependent fear memory (Twining et al., 2020), renewal of fear (Wang et al., 2016; Marek et al., 2018; Szadzinska et al., 2021), and anxiety-related behavior (Adhikari et al., 2010; Padilla-Coreano et al., 2016). In contrast, the dorsal circuits connecting dMFC with dcHPC are probably involved in spatial-related cognitive functions, as dcHPC likely provides spatial information of different nature compared with proximal CA1 (Xiao et al., 2020; Deshmukh, 2021). Furthermore, some reports showed that dcHPC may modulate nociceptive responses in PL and AC (Nakamura et al., 2010) and even strengthen fear memories through PL (Ye et al., 2017).

Although the MFC-HPC circuits seem functionally segregated along the dorsoventral axis—dorsal for spatial cognition and ventral for emotion—it is likely that these circuits work together to support episodic memory processes (Eichenbaum, 2017; Pronier et al., 2023). MFC clearly requires some awareness of current spatial information to enable behaviors such as decision-making and flexibility. While intrinsic connectivity within either HPC (Messanvi et al., 2022) and/or MFC may facilitate this interplay, our results point to an additional, unknown direct pathway, connecting dcHPC to the most ventral part of vMFC, potentially enhancing such communication (Fig. 9*B*).

### The dcHPC-vMFC circuit as a connection between dorsal–caudal and ventral HPC-MFC

In addition to the parallel ventral and dorsal circuits described above, we propose a third circuit consisting of a “crossed” connectivity along the dorsoventral axis, by which dcHPC sends monosynaptic inputs to the ventral-most MFC, i.e., DP and MO, but projects disynaptically back to vHPC (Fig. 9*B*).

Connections of DP with the rest of the brain are comparable to IL (Gabbott et al., 2005). However, DP has specific connections that suggest that within the MFC subregions, DP may have a different functionality (Rodgers et al., 2008; Akhter et al., 2014; Gretenkord et al., 2017; Mathiasen et al., 2023). Particularly, DP projects densely to the mammillary body (Mathiasen et al., 2019) and receives dense inputs from LEC (Insausti et al., 1997). Additionally, robust specific spatial-related information may reach MFC through this dcHPC-DP circuit (Burke et al., 2011; Xiao et al., 2020; Kitanishi et al., 2021). Recent studies suggest that DP plays an important role in autonomic responses and emotional-related behavior, including driving sympathetic stress responses through dorsomedial hypothalamus connections (Kataoka et al., 2020), regulating fight-or-flight responses via central AMG connectivity (Borkar et al., 2024), and modulating affective behavior and fear memory (Botterill et al., 2024; Campos-Cardoso et al., 2024). This dcHPC-DP circuit may therefore be essential for memory processing, emotional-related behavior, and autonomic/sympathetic responses.

Whereas the DP cortex is primarily innervated by dcHPC, MO receives inputs from both vHPC and dcHPC. Evidence suggests that MO plays an important role in mediating outcome predictions for goal-directed behavior (Bradfield et al., 2015). In line with this function, vHPC-MO projections may support flexible behavior by facilitating predictions about future events (Wikenheiser and Schoenbaum, 2016; Woon et al., 2022). However, the specific contribution of dcHPC projections to MO remains unclear.

## Conclusion

Our findings reveal a dorsoventral organization of HPC-MFC projections, involving two parallel pathways from dcHPC and vHPC, targeting distinct excitatory and inhibitory neuron populations in MFC. The direct HPC-MFC projections and indirect MFC-HPC projections form two circuits (Fig. 9): a known reciprocal circuit connecting the emotional-related areas vHPC and vMFC (Fig. 9*A*) and a newly identified circuit connecting the cognition-related dcHPC with the emotion-related vMFC, particularly the DP cortex, which projects back to vHPC (Fig. 9*B*). This highlights a functional heterogeneity along the dorsoventral axis of both HPC and MFC, with potential implications for memory processing, emotional regulation, and related neurological and psychiatric disorders.
